# RNA exosome ribonuclease DIS3 degrades *Pou6f1* to promote mouse pre-implantation cell differentiation

**DOI:** 10.1016/j.celrep.2023.112047

**Published:** 2023-01-30

**Authors:** Di Wu, Jurrien Dean

**Affiliations:** 1 Laboratory of Cellular and Developmental Biology, NIDDK, National Institutes of Health, Bethesda, MD 20892, USA; 2 Lead contact

## Abstract

Mammalian development is precisely controlled by cell differentiation. Identifying new regulators and investigating their interactions provide insight into genetic networks defining pre-implantation development. We established a knockout mouse model of *Dis3*, an exosome associated ribonuclease. Homozygous *Dis3* null embryos arrest at the morula stage of development. Using single-embryo RNA sequencing (RNA-seq), we observed persistence of *Pou6f1* mRNA in homozygous null *Dis3* embryos and that the cognate protein represses transcription of *Nanog* and *Cdx2*. The resultant defects in cell differentiation disrupt the morula-to-blastocyst transition and are embryonic lethal. Microinjection of *Dis3* mRNA into zygotes rescues the phenotype. Point mutations of *Dis3* ribonuclease in individual blastomeres prevents their incorporation into embryos. To overcome the paucity of embryos, we derived homozygous *Dis3* null mouse embryonic stem cells to identify additional gene targets of POU6F1. Our findings delineate a regulatory pathway of DIS3-POU6F1 in pre-implantation mammalian embryogenesis.

## In brief

Mammalian pre-implantation development is regulated by master transcription factors. Wu and Dean report that an RNA-exosome-associated ribonuclease, DIS3, degrades *Pou6f1* mRNA to de-repress *Nanog* and *Cdx2* transcription. In the absence of DIS3, POU6F1 persists, and mouse embryos arrest as morulae and are unable to become blastocysts due to deficient differentiation.

## Graphical abstract

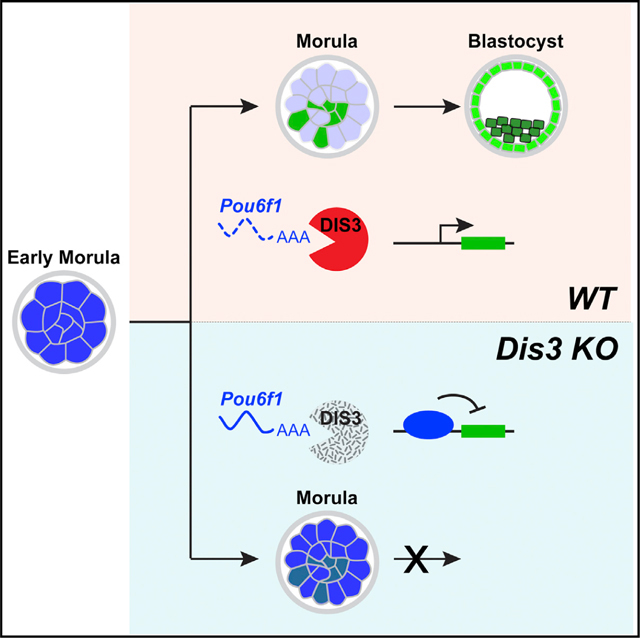

## INTRODUCTION

Sequential cell fate specification occurs during cell cleavage in early mammalian development and is controlled by both maternal and zygotic factors. The maternal-to-zygotic transition in early mammalian development is conserved and includes maternal RNA degradation as well as zygotic gene activation (ZGA).^1,2^ Failure to clear maternal RNAs and/or activate zygotic genes result in embryonic arrest.^3,4^ Loss-of-function studies document critical licensing factors that control zygotic gene transcription,^2^ but less is known about the molecular regulation governing clearance of repressive transcripts.

The RNA exosome complex is responsible for degrading RNA in many different cell types. The well-conserved exosome complex binds to associated ribonucleases (DIS3, EXOSC10, DIS3L) to acquire enzymatic activity.^5,6^ One of the most important RNA-exosome-associated ribonucleases, DIS3, is essential for cell survival and embryo development in flies.^7^ Depletion of DIS3 causes accumulation of a wide range of transcripts, including protein-coding, non-coding, and repetitive element RNAs. Using cross-linking immunoprecipitation sequencing (CLIP-seq), the direct RNA targets of DIS3 have been reported in mammalian cultured cell lines.^8,9^ Although target selection of DIS3 lacks sequence specificity, the binding profiles exhibit strong tissue preference because of distinct transcriptomes in different cells.^7–10^ Thus, preventing RNA degradation by DIS3 depletion can identify regulators of development when their persistence causes significant downstream effects.

During mammalian pre-implantation development, the embryo undergoes ZGA, cell cleavage, compaction, cavitation, and differentiation.^11^ At the end of the blastocyst stage, three cell lineages are defined by position and expression of unique markers, namely epiblast (EPI; expressing NANOG), primitive endoderm (PrE; expressing GATA4/GATA6), and trophectoderm (TE; expressing CDX2/GATA3).^12^ Master transcription factors, including SOX2, OCT4, NANOG, and CDX2,^13–15^ drive serial differentiation during two sequential cell fate decisions. The first differentiates the inner cell mass (ICM) from the TE, and the second differentiates the EPI from the PrE within the ICM.^12^ The mutual activation and repression between factors synchronize different cell lineages for future germ layers. For example, OCT4 and SOX2 directly initiate *Nanog* transcription,^16^ and *Nanog* and *Cdx2* mutually repress each other’s transcription by occupying promoter regions.^17^ Although the molecular mechanism underlying the dynamic sculpting of different cell fates has been explored extensively, the understanding of its complexity remains incomplete.

In this study, we constructed *Dis3* ribonuclease knockout mice and discovered homozygous embryos arrest during pre-implantation development. We demonstrate that defects occur in cell differentiation during the morula-to-blastocyst transition. Through single-embryo RNA-seq analysis, we identified POU6F1, a transcription repressor of *Nanog* and *Cdx2*, as a potential target for DIS3-mediated degradation. We profiled gene targets of POU6F1 to confirm its regulatory activity on transcription. Finally, we tested the effects of mutant forms of DIS3, which provide insights into formation of congenital developmental abnormalities and oncogenesis.

## RESULTS

### *Dis3* homozygous null embryos arrest at the morula-to-blastocyst transition

**Figure 1.**
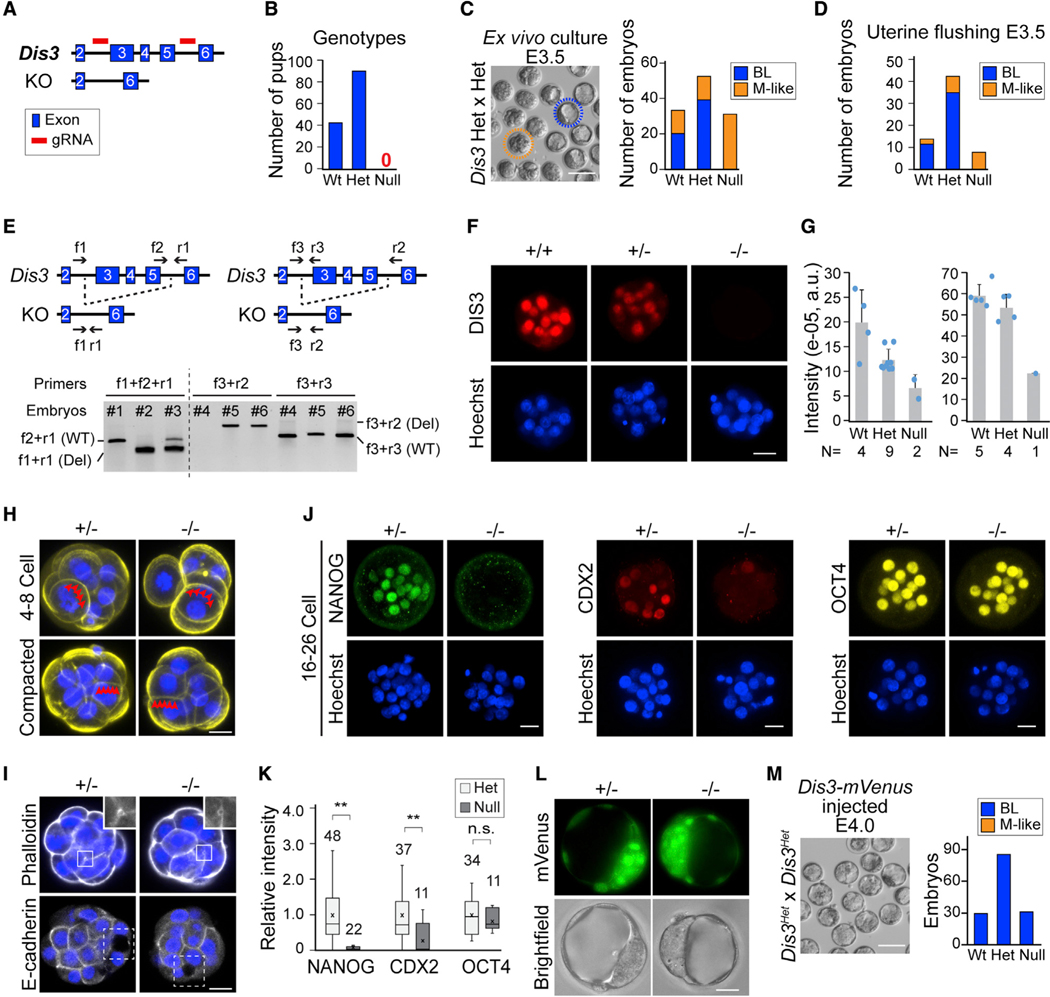
*Dis3* null embryos arrest at the morula-to-blastocyst transition and lack cell differentiation (A) Schematic of *Dis3* knockout mice construction. Exons 3–5 are absent in the *Dis3* null allele. (B) Bar graph showing the number of pups of each genotype born from *Dis3* heterozygous mating. (C and D) Number of blastocysts (BLs) and morula-like (M-like) embryos in *ex vivo* culture (C) and uterine flushing (D) at E3.5 derived from *Dis3* heterozygous matings. (E) Schematic of the null allele and genotyping. Two genotyping strategies are illustrated. Arrows indicate the positions of primers used. In the first strategy (left), f2+r1 are specific to the wild-type (WT) allele, and f1+r1 are specific to the null allele. In the second strategy (right), f3+r3 are specific to the WT allele, and f3+r2 are specific to the null allele. #1–6, six embryos: #1 and #4, WT; #2, homozygous null; #3, #5, and #6, heterozygous. (F and G) Immunofluorescence and quantification of DIS3 in WT (+/+) and *Dis3* heterozygous (+/−) and homozygous (−/−) null embryos. The two bar graphs are from two representative assays. Each bar graph shows embryos derived from one *Dis3* heterozygous female after mating to a *Dis3* heterozygous male. The number of embryos is indicated below each bar. a.u., arbitrary unit. (H) Phalloidin staining of *Dis3* heterozygous (+/−) and null (−/−) embryos at the 4- to 8-cell stage before and after compaction. Red triangles indicate boundaries of blastomeres. (I) Immunofluorescence of heterozygous (+/−) and homozygous (−/−) null embryos after staining with phalloidin and E-cadherin. Insets, regions enlarged to show the microlumen. (J and K) Immunofluorescence and quantification of NANOG, CDX2, and OCT4 in *Dis3* heterozygous (+/−) and homozygous (−/−) null embryos. **NANOG, p = 2.2E−10; **CDX2, p = 0.0006; n.s., not significant; two-tailed Student’s t test. (L) mVenus fluorescence of the injected *Dis3-mVenus* mRNA in *Dis3* heterozygous (+/−) and homozygous (−/−) null embryos. (M) Number of BL and M-like embryos in *ex vivo* culture at E3.5 of embryos derived from *Dis3* heterozygous mating and microinjected with *Dis3-mVenus* mRNA at 1-cell stage. Scale bars, 100 μm in (C) and (M) and 20 μm in (F), (H)–(J), and (L).

During pre-implantation, DIS3 ribonuclease is expressed from the 4-cell stage onward, suggesting its possible regulatory role in embryo cleavage and fate decision (Figure S1A). A *Dis3* null allele was fortuitously generated while establishing mouse lines with a floxed allele. Exons 3–5 were deleted by CRISPR-Cas9 and imprecisely repaired through non-homologous end joining (Figures 1A, 1E–1G, and S3C) to create the null allele. We crossed heterozygous (het) *Dis3* mice, and none of the pups born were homozygous null for *Dis3*, suggesting embryonic lethality (Figure 1B). In contrast, *Dis3* het mice were healthy (Figures 1B and S1B), grew to adulthood, and were fertile.

We sought to identify the stage of lethality of *Dis3* null embryos. In an *ex vivo* culture of the pre-implantation embryos produced by *Dis3* het mating, we observed a decrease of viability at embryonic day 4 (E4) (Figure S1C). Around 25% of the embryos arrested at the morula stage and could not form a blastocoel. We defined the embryos as either blastocysts (BLs; having an obvious blastocoel) or morula (M)-like (not having an obvious blastocoel) at E3.5 and performed single-embryo genotyping. All *Dis3* homozygous null embryos belonged to the M-like group and did not progress developmentally, even after extended culture (Figure 1C). The same M-arrest phenotype could be recapitulated by uterine flushing of female mice at E3.5 after *Dis3* het mating (Figure 1D). To further validate the arrest, we synthesized *Dis3* morpholino (MO2) and microinjected it into 1-cell wild-type embryos to block translation of *Dis3* mRNA (Figures S1D and S1E). As expected, *Dis3* morpholino efficiently blocked the translation of its downstream reporter gene, and *Dis3* morpholino injection increased M-like embryos and reduced BLs at E4.0 in a dose-dependent manner (Figures S1F–S1H).

To further investigate the cause of M arrest, we confirmed that cells did not undergo apoptosis in *Dis3* null embryos at E3.5 (Figures S2A and S2B). By outlining cell borders with phalloidin, we observed successful compaction in *Dis3* null embryos (Figure 1H). *Dis3* null embryos also initiated blastocoel cavitation by forming microlumens visualized by phalloidin and E-cadherin immunostaining (Figure 1I). In addition, *Dis3* null embryos did not have obvious differences in their endomembrane systems (Figures S2C). However, when we performed immunostaining of NANOG and CDX2, which are markers of cell specification, homozygous *Dis3* null embryos had significantly reduced levels of NANOG and CDX2 (Figures 1J, 1K, and S2D), which was not a reflection of embryonic stage. Another differentiation marker, OCT4, remained normal in early *Dis3* null embryos due to its maternal origin (Figures 1J and 1K). To further document that DIS3 was essential for the M-to-BL transition, we synthesized *Dis3-mVenus* mRNA *in vitro* and microinjected it into the 1-cell embryos derived from *Dis3* het mating. Overexpression of *Dis3-mVenus* mRNA resulted in an increase in the number of BLs because the homozygous *Dis3* null embryos successfully formed a blastocoel (Figures 1L and 1M). To summarize, *Dis3* null embryos arrest at M-to-BL transition due to defective cell differentiation.

### DIS3 degrades *Pou6f1* mRNA to de-repress *Nanog* and *Cdx2* transcription

**Figure 2.**
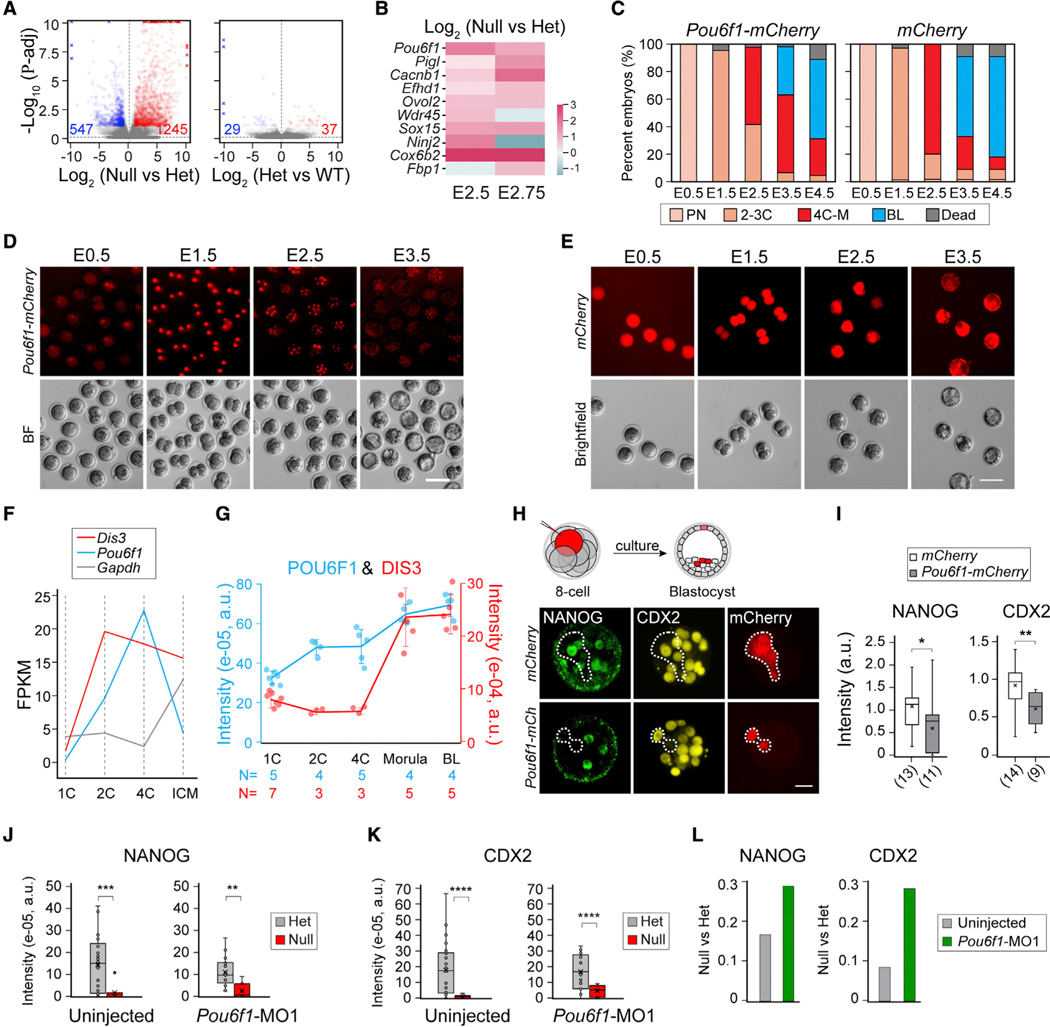
*Pou6f1* is up-regulated in *Dis3* null embryos and blocks *Nanog* and *Cdx2* transcription (A) Volcano plots showing differentially expressed genes from single-embryo RNA-seq of combined analyses of E2.5 and E2.75. Left, *Dis3* homozygous vs. heterozygotes (het) embryos (null vs. het); right, *Dis3* het vs. WT (het vs. WT). Blue and red, significantly down- and up-regulated genes, respectively (adjusted p value [p-adj] < 0.1). (B) Heatmap showing the change of 10 selected genes in null vs. het at E2.5 and E2.75. (C) Bar graph showing the ratio of embryos at each stage from E0.5 to E4.5 in *ex vivo* culture. WT 1-cell embryos (around 20) were injected either with *Pou6f1-mCherry* (n = 21) or *mCherry* (n = 19). Results combine two assays. (D and E) Fluorescent and bright-field images of embryos from E0.5 to E3.5 that were either *Pou6f1-mCherry* injected or *mCherry* injected. (F) Fragments per kilobase million (FPKM) of *Pou6f1, Dis3*, and *Gapdh* from GEO: GSE57249 RNA-seq results of blastomeres at different stages of mouse preimplantation development. (G) Quantification of POU6F1 (blue) and DIS3 (red) proteins by immunofluorescence at 1C, 2C, 4C, M, and BL stages. The number of embryos is indicated below each stage. (H and I) Schematic, immunofluorescence staining, and quantification of NANOG and CDX2 in blastomeres injected with *Pou6f1-mCherry* or *mCherry* (in mCherry-positive cells). *NANOG, p = 0.096; **CDX2, p = 0.009. (J and K) Quantification of NANOG and CDX2 immunofluorescence in *Dis3* het and homozygous embryos (null) at E3. Embryos are either uninjected or microinjected with *Pou6f1*-MO1 0.5 mM at the 1C stage. ***p = 0.00011 in (J), **p = 0.0014 in (J), ****p = 3.1E–5 in (K), left, ****p = 5.3E–5 in (K), right. (L) Bar graph showing the ratio of NANOG and CDX in *Dis3* null vs. het embryos. The value of the bar is calculated by the mean values of each group from (J) and (K). Scale bars, 20 μm in (H) and 100 μm in (D) and (E).

The reduction of NANOG and CDX2 suggested an inhibition of their gene transcription in homozygous *Dis3* null embryos. We hypothesized that depletion of DIS3 ribonuclease results in insufficient degradation of mRNA encoding a repressor, which blocked gene transcription prior to the morula stage. To profile potential DIS3 target transcripts, we performed single-embryo RNA-seq at E2.5 and E2.75. As expected, *Dis3* het and wild-type embryos were grouped together by principal-component analysis (PCA), whereas homozygous *Dis3* null embryos at both stages were in a distinct group (Figure S3A). Due to the similarity of the differential genes at the two stages, we combined them for a differential analysis. As a result, we obtained 1,245 up-regulated genes and 547 down-regulated genes in the null vs. het comparison and only 37 up-regulated and 29 down-regulated genes in the het vs. wild-type comparison (Figure 2A). The decrease of *Nanog* and *Cdx2* transcripts was also apparent in the differential analyses (Table S2). In addition to coding RNAs, PROMPTs RNA, which are known DIS3 targets, also exhibited significant accumulation (Figure S3B).^8,9^

To screen for DIS3 targets, we took advantage of published DIS3 CLIP-seq datasets from cultured cell lines to overlap with the protein-coding genes that were significantly increased in at least one stage in our RNA-seq results (Figures 2B and S3D).^9^ From the obtained gene lists, we synthesized 10 mRNAs *in vitro* as mCherry fusion transcripts and overexpressed the mRNAs individually by microinjection into 1-cell wild-type embryos (Figures 2C–2E and S4A). We observed that *Pou6f1* overexpression increased morula arrest at E3.5, and consequently, there were fewer BLs at E4.5 (Figures 2C–2E). From public datasets (GEO: GSE57249 and GSE45719), we confirmed that RNA levels of *Pou6f1* increased in wild-type embryos as they advanced from the 1- to 4-cell stage and then decreased in the BL stage^18,19^ (Figures 2F and S3F). The cognate protein increased slowly from 1C to morula stages, and the accumulation stopped after morulae (Figure 2G), which suggested a need for POU6F1 clearance when getting close to the BL stage. On the other hand, the *Dis3* mRNA level increased from the 1C stage, followed by a DIS3 protein surge at the 4C-M stage, which preceded *Pou6f1* RNA degradation (Figures 2F, 2G, and S3F). Degradation of *Pou6f1* transcripts was prevented in homozygous *Dis3* null embryos, although the increase of POU6F1 protein was not statistically significant in homozygous *Dis3* null embryos due to the embryonic variation and the reduced embryonic quality upon DIS3 depletion (Figures 2B and S5E).

To validate the inhibitory effect of POU6F1 on *Nanog* and *Cdx2* transcription, we microinjected *Pou6f1-mCherry* mRNA into 1–2 blastomeres at the 8-cell stage and measured NANOG/CDX2 at the BL stage by immunostaining (Figure 2H). As expected, cells injected with *Pou6f1-mCherry* mRNA had lower NANOG and CDX2 intensities compared with *mCherry*-injected control cells (Figure 2I). Then, we synthesized *Pou6f1* morpholino (MO1) to knock down *Pou6f1* after microinjection into embryos. We determined 0.5 mM *Pou6f1* MO1 as the most effective and non-toxic concentration because it did not cause significant embryonic lethality (Figure S5A). Microinjection of *Pou6f1* MO1 reduced POU6F1 protein level (Figures S5B and S5C). However, the morula arrest phenotype of *Dis3* null embryos could not be rescued by *Pou6f1* MO1 (Figure S5D). Nevertheless, when we examined the level of NANOG and CDX2 in *Pou6f1* MO1-injected *Dis3* null embryos, the level was increased though still less than their level in the *Dis3* het embryos (Figures 2J–2L). The partial rescue effect of *Pou6f1* MO1 further supports the role of POU6F1 in repressing cell specification to cause morula arrest of *Dis3* null embryos and indicates that other factors participate in this process. We conclude that homozygous *Dis3* null embryos fail to degrade *Pou6f1*, which consequently repressed transcription of *Nanog* and *Cdx2* genes.

### POU6F1 binds to gene promoters and represses transcription in mouse embryonic stem cells

**Figure 3.**
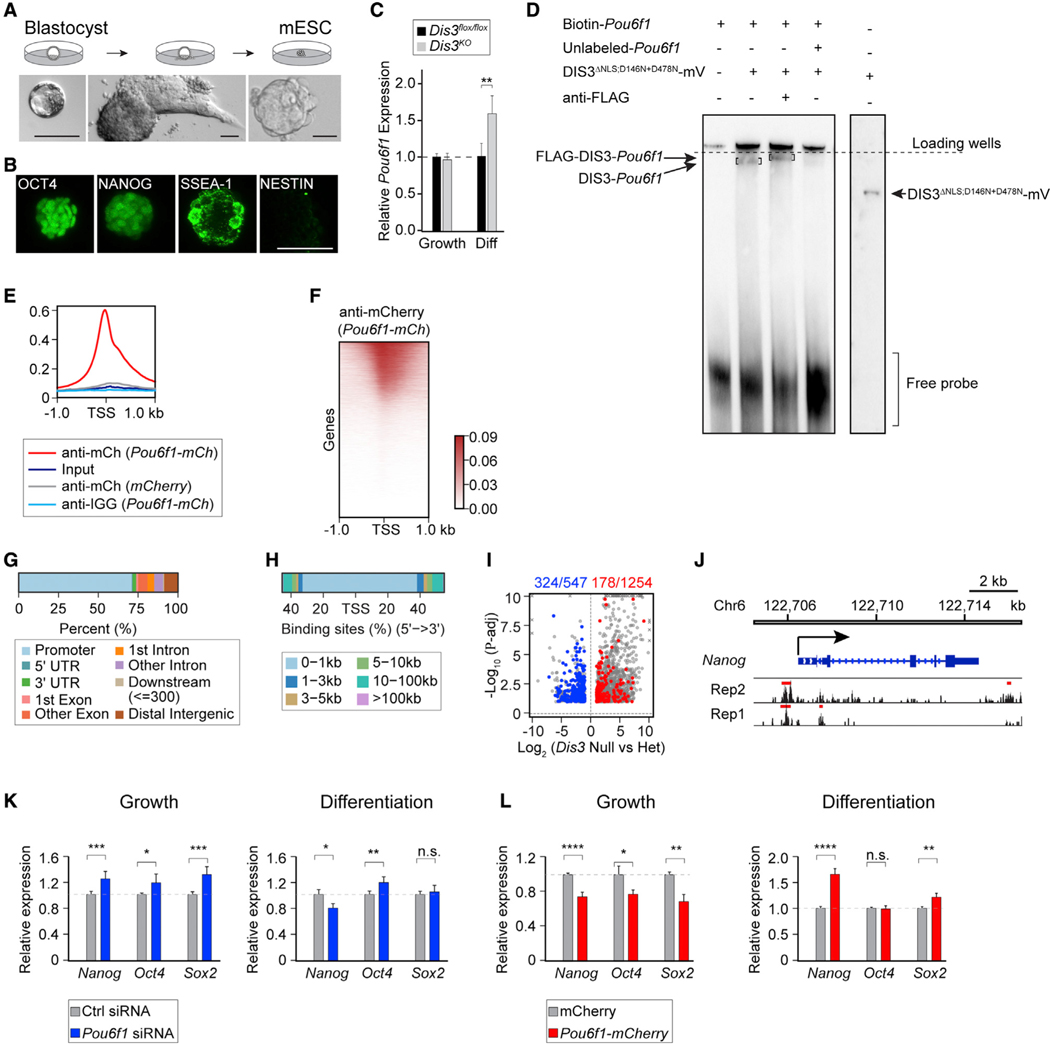
POU6F1 globally occupies promoters to regulate gene transcription in mouse embryonic stem cells (A) Schematic of mouse embryonic stem cell (mESC) derivation from *Dis3*^*flox/flox*^ BLs. (B) Immunofluorescence staining of OCT4, NANOG, SSEA-1, and NESTIN in mESCs. (C) Bar graph showing *Pou6f1* changes in growth and differentiation (Diff) phases of mESCs after knockout of *Dis3*. **p = 0.01. (D) Electrophoretic mobility shift assay (EMSA) result showing DIS3^ΔNLS;D146N+D478N^-mVenus protein binding to *Pou6f1* RNA. D146N+D487N, double mutation of DIS3. ΔNLS, nuclear localization signal peptide deleted, as described in Figure 4. On the right shows the protein band detected in immunoblot using anti-FLAG (engineered epitope in the mVenus protein). Two results are aligned by the positions of loading wells and loading dye. (E and F) Profile and heatmap of POU6F1 occupancy from ChIP-seq analysis. (G and H) Distribution of POU6F1 occupied genome loci in different genomic regions and the relative distances of the peaks to TSSs. (I) Volcano plot of significantly changed genes from RNA-seq (from Figure 2A) having POU6F1 occupancy. Blue and red, significantly changed down- and up-regulated genes in *Dis3* null vs. het that are occupied by POU6F1. (J) POU6F1 peaks on *Nanog*. Rep1 and 2: two biological replicates. Red bars: peaks called against anti-immunoglobulin G (IgG) samples. (K) qRT-PCR of *Nanog*, *Oct4*, and *Sox2* by POU6F1 knockdown using *Pou6f1* siRNA transfection. Cells are in the growth condition (left) or differentiation condition (right). ***Nanog, 0.0007, *Oct4, 0.02, ***Sox2, 0.0002 in growth condition; *Nanog, 0.03, **Oct4, 0.007, n.s., not significant in differentiation condition. (L) qRT-PCR of *Nanog*, *Oct4*, and *Sox2* by POU6F1 overexpression using *Pou6f1-mCherry* transfection. Cells are in the growth condition (left) or differentiation condition (right). ****Nanog, 7e-05, *Oct4, 0.02, **Sox2, 0.007 in growth condition; ****Nanog, 3e-05, n.s., not significant, **Sox2, 0.004 in differentiation condition. Scale bars, 100 μm in (A) and 50 μm in (B).

**Figure 4.**
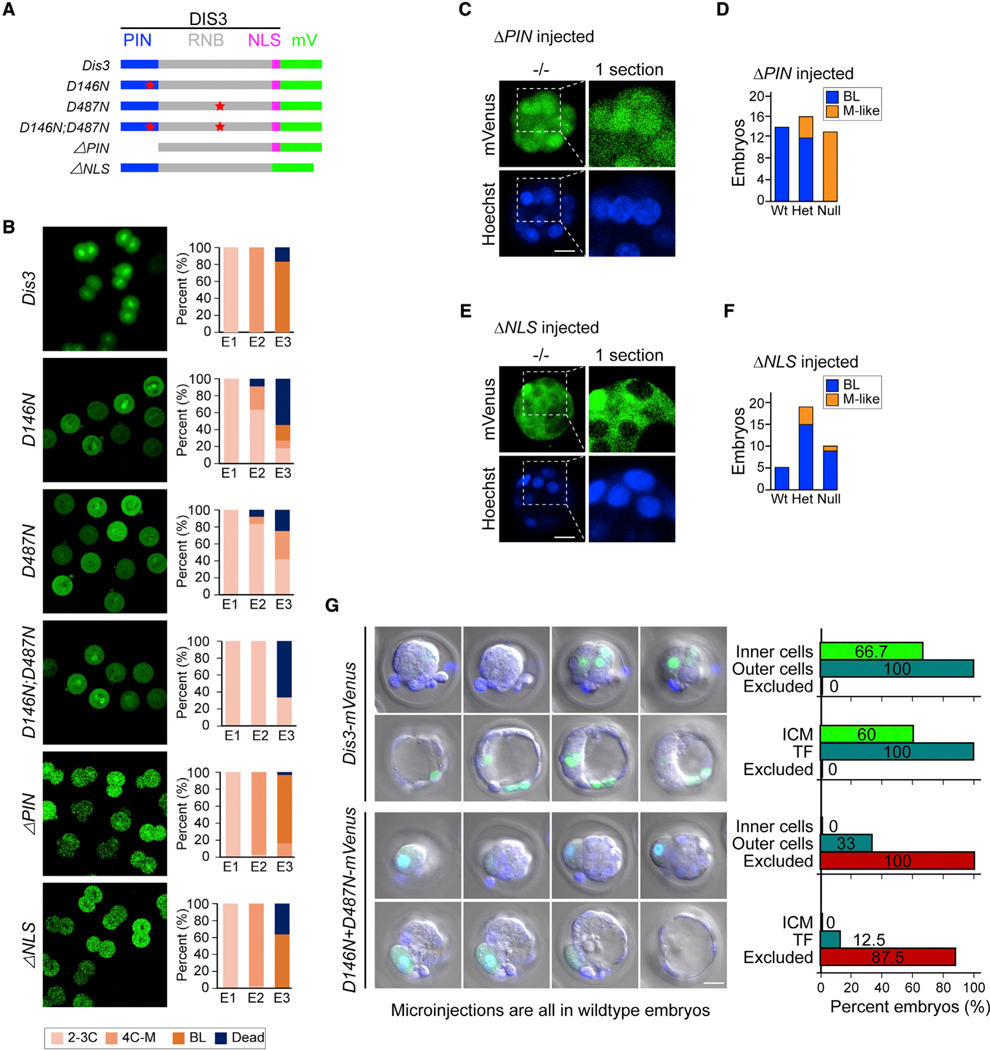
Mutant forms of DIS3 can induce cell exclusion in early embryonic development (A) Schematic of all DIS3 mutant forms of protein. PIN: DIS3 PIN domain; RNB: DIS3 RNB domain; NLS: DIS3 nuclear localization signal; mV: mVenus coding region. (B) Fluorescent live imaging of DIS3 mutant proteins and the ratio of embryos at different stages from E1 to E3. All proteins have a mVenus tag at the C terminus as illustrated in (A). (C) Fluorescent live imaging of DIS3^ΔPIN^-mVenus protein at the BL stage in *Dis3* null embryos. Embryos were microinjected with *Dis3*^*ΔPIN*^*-mVenus* mRNA, cultured, imaged individually, and collected for single-embryo genotyping. The whole embryo picture is a z-projection of optical sections. The boxed zone is magnified on the right, and only 1 optical section is shown to visualize the fluorescence localization. (D) Ratio of BL and M-like embryos from *Dis3* heterozygous mating and microinjected with *Dis3*^*ΔPIN*^*-mVenus* mRNA. (E) Same as (C) but after microinjection of *Dis3*^*ΔNLS*^*-mVenus* mRNA. (F) Same as (D) but after microinjection of *Dis3*^*ΔNLS*^*-mVenus* mRNA. (G) Fluorescent live imaging and cell position quantification after microinjection with *Dis3-mVenus* or *Dis3*^*D146N+D487N*^*-mVenus* mRNA into 1–2 blastomeres at the 4- to 8-cell stage of WT embryos. Cell positions were visually identified as inner, outer, and excluded based on their position and size. Fluorescent marker is mVenus of the injected mRNA encoding the fusion protein. Scale bars, 100 μm in (B) and 20 μm in (C), (E), and (G).

Our results suggest possible roles of DIS3 ribonuclease and POU6F1 in regulating gene expression and cell fate transition. To directly test this hypothesis, we derived mouse embryonic stem cells (mESCs) from *Dis3^flox/flox^* BLs (Figure 3A).^10^ The integrity of the derived mESCs was confirmed by the presence of the stem cell markers OCT4, NANOG, and SSEA-1 and the absence of the differentiation marker NESTIN (Figure 3B). mESCs maintained stemness in self-renewal medium and underwent differentiation upon removal of self-renewal factors (Figures S6A and S6B). The differentiated mESCs became negative for NANOG and positive for NESTIN as well as the neural ectodermal marker PAX6 (Figure S6C). When DIS3 was ablated by transfecting *Cre* recombinase with the cytomegalovirus enhancer fused to the chicken β-actin (CAG) promoter into the mESCs, we observed a significant up-regulation of *Pou6f1* when cells were differentiated (Figure 3C). To validate the ability of DIS3 ribonuclease to bind *Pou6f1* mRNA, we synthesized the DIS3-occupied fragment of *Pou6f1* from the published CLIP-seq dataset to perform electrophoretic mobility shift assay (EMSA)^9^ (Table S1; Figure S3D). A mutant form of the DIS3 protein (D146N+D487N, defined below) was used to avoid RNA degradation, and the nuclear localization signal (NLS) was deleted because the cytoplasmic function of DIS3 is sufficient to support pre-implantation development (DIS3^ΔNLS;D146N+D487N^-mVenus; Figures 3D, 4A, and 4B). As a result, the DIS3 ribonuclease protein bound to the synthesized *Pou6f1* fragment, which was further validated by generating a super-shifted complex after the addition of anti-FLAG antibody recognizing mVenus protein (Figure 3D).

Next, we performed chromatin immunoprecipitation (ChIP)- seq of POU6F1 in mESCs to profile target genes. Two biological replicates exhibiting high reproducibility were combined for analysis of POU6F1-binding sites (Figures S6D and S6E; Table S3). Overall, we identified ~18,000 peaks (Table S3) as POU6F1-binding sites that were close to transcription start sites (TSSs) (Figures 3E and 3F). More than 70% of these peaks were present in promoter regions (Figure 3G), and more than 80% of the peaks were within 1 kb of the TSS (Figure 3H). When comparing POU6F1-occupied genes with the differentially expressed transcripts from the *Dis3* embryonic RNA-seq, ~60% (324/547) of the down-regulated genes were occupied by POU6F1, while only ~15% (178/1,254) of the up-regulated genes were occupied by POU6F1 (Figure 3I). From the POU6F1-occupied promoters, there were 317/9,119 genes involved in *in utero* embryonic development (q = 1.5E–26; Table S4). Specifically, we observed occupancy of POU6F1 in *Nanog* and *Cdx2* promoters and genic regions (Figures 3J and S6F).

To further examine the possible repressive function of POU6F1 on stem cell marker genes, we performed *Pou6f1* knockdown using small interfering RNA (siRNA) transfection and *Pou6f1* overexpression using *Pou6f1-mCherry* transfection (Figures S6G and S6H). Knockdown of *Pou6f1* led to increased expression of *Nanog*, *Oct4*, and *Sox2*, whereas its overexpression caused reduced levels of the mESC marker genes (Figures 3K–3L). The repressive role of POU6F1 on mESC marker genes was less significant when mESCs underwent differentiation, suggesting that the regulatory role of POU6F1 is cell-context dependent (Figures 3K–3L). In summary, *Pou6f1* mRNA is degraded by DIS3 ribonuclease, and its persistence in the absence of DIS3 is associated with continued repression of developmental master regulators with subsequent embryonic arrest.

### Mutant DIS3 ribonuclease proteins induce cell exclusion

Mutations of DIS3 ribonuclease have been reported in several human diseases, including multiple myeloma and acute myeloid leukemia.^20,21^ Two highly conserved enzymatic sites have been identified in DIS3 protein. D146 is essential for the PIN endonuclease activity by positioning the metal ion required for cleavage activity,^22^ while D487 is essential for the RNB exonuclease domain activity.^23^ Each single mutation can cause transcriptome disruption due to their dominant-negative effect, and double mutations can lead to much more significant perturbations to the transcriptome in human cell lines.^9^

We engineered the wild-type DIS3 protein into several mutant isoforms, including single loss-of-function point mutation (DIS3^D146N^, DIS3^D487N^), a double mutation (DIS3^D146N+D487N^), an endonucleolytic deletion (DIS3^ΔPIN^), and a NLS deletion (DIS3^- Δ^NLS) (Figure 4A). After microinjection into 1-cell wild-type embryos, *Dis3^D146N^*, *Dis3^D487N^*, and *Dis3^D146N+D487N^* mRNA caused embryonic arrest at the 2-cell stage (Figure 4B). However, overexpression of *Dis3^ΔPIN^* and *Dis3^ΔNLS^* did not affect pre-implantation development (Figure 4B). We then tested whether *Dis3^ΔPIN^* and *Dis3^ΔNLS^* overexpression could rescue *Dis3* null embryos. *Dis3^ΔPIN^* mRNA failed to rescue, suggesting that the cognate protein was non-functional and non-toxic (Figures 4C and 4D). Unexpectedly, *Dis3^ΔNLS^* overexpression successfully rescued *Dis3* null embryos to form BLs, which further supports a model in which the lack of cytoplasmic, rather than nuclear, DIS3 function was more likely to cause the morula arrest (Figures 4E and 4F).

To determine the effect on single blastomeres in embryos, we microinjected wild-type *Dis3* or *Dis3^D146N+D487N^* mRNA into 1–2 blastomeres at the 8-cell stage of wild-type embryos and recorded the position and morphology of the injected blastomeres at later stages. Blastomeres injected with wild-type *Dis3* mRNA were present in either inner or outer positions in morulae and blastocysts (Figure 4G). In contrast, blastomeres injected with *Dis3^D146N+D487N^* mRNA were all restricted to the embryo surface (Figure 4G). They did not appear as “outer cells” because their sizes were significantly larger than uninjected cells in the same embryo. Instead, we defined them as excluded cells due to their inability to incorporate into dividing embryos, which may be due to transcriptome disruption and/or concurrent cell division defects.^24^ We conclude that expression of mutant DIS3^D146N+D487N^ directly results in embryonic arrest or cell exclusion in pre-implantation development.

## DISCUSSION

Our results document the essential role of RNA degradation in early embryo development. Deletion of *Dis3*, an exosome-associated ribonuclease, results in developmental arrest at the M-to-BL transition. This led to the identification of *Pou6f1* mRNA as an important target of DIS3 ribonuclease at this stage of development. The persistence of *Pou6f1* mRNA due to insufficient degradation leads to more abundant POU6F1 protein, which represses transcription of *Nanog* and *Cdx2* and blocks cell specification.

Mutual cooperative interactions and/or antagonistic regulation of master transcription factors is a hallmark of sequential cell fate specification that defines cell lineage. A transcription regulator can not only prime cells for downstream differentiation but also represses their proclivity toward other cell fates. For example, OCT4 (POU5F1) activates *Nanog* transcription in future ICM cells and represses *Cdx2* transcription to prevent a TE fate.^17,25^ In contrast, in future TE cells, *Oct4* and *Nanog* are repressed by CDX2.^26^ In the current study, we identify POU6F1, another member of the POU-homeobox domain family transcription factors, as actively involved in this regulatory network. POU6F1 represses both *Nanog* and *Cdx2* gene expression at the morula stage in wild-type embryos, which blocks cell fate specification. If *Pou6f1* mRNA is not degraded, cells cannot differentiate, and embryos arrest as morulae during pre-implantation development. Similar observations were made in mESCs cultured *in vitro*. The up-regulated genes in *Dis3* null embryos that are occupied by POU6F1 could be either caused by POU6F1 acting as an activator by itself or in combination with other regulators. In addition, there are also many more coding and non-coding genes dysregulated in *Dis3* null embryos than *Pou6f1*, which could also cause direct or indirect changes on RNA degradation and transcription. A possible cell-context-specific role of POU6F1 is also suggested from the different inhibitory roles on the mESC marker genes in growth and differentiation conditions: (1) POU6F1 may be required for additional genes other than *Nanog* during cell fate transition, and (2) conversely, the level of POU6F1 could be only one of the many regulators of *Nanog* repression. Consistent with these results, POU6F1 has also been reported to be essential for specification and patterning of dendrites, and its overexpression can promote dendritic outgrowth and branching.^27^ POU6F1 potentially interacts with ERBB2 and NFYC as indicated in two public databases, BioGRID^28^ and IntAct.^29^ Both ERBB2 and NFYC are nuclear proteins and have known target genes.^30,31^ POU6F1, ERBB2, and NFYC shared many binding targets, suggesting a potential cooperation of POU6F1 with the other factors in regulating the target gene’s transcription.

Our findings demonstrate that zygotic DIS3 ribonuclease is essential for the M-to-BL transition during pre-implantation development. The level of *Dis3* transcript is decreased in *Dis3* null embryos from E2.5 onward. However, these results do not rule out a function for DIS3 ribonuclease before E2.5 due to maternal persistence of *Dis3* RNA and protein. To address this issue, we synthesized a *Dis3* morpholino to block maternal RNA translation, which could expose an earlier phenotype by reducing the maternal DIS3 ribonuclease protein level. However, we obtained the same morula arrest phenotype in *Dis3* MO-injected embryos, which suggests that maternal *Dis3* RNA is not essential for early embryogenesis. Whether maternal DIS3 protein regulates pre-implantation development remains unknown because conditional depletion of DIS3 ribonuclease in oocytes blocks oocyte maturation and precludes studying a post-fertilization maternal effect.^10^

The RNA exosome complex is responsible for degrading many types of RNA. Multiple studies have focused on finding a sequence-based affinity of DIS3-bound RNAs without success, suggesting that the recognition and degradation by DIS3 does not depend on sequence-defined domains.^8,9^ We reasoned that one or more persistent transcripts could cause a particular phenotype but only under certain cellular conditions or in combination with other perturbations. Indeed, among the 10 genes we screened from the up-regulated transcripts in *Dis3* null embryos, only *Pou6f1* overexpression partially phenocopied the M arrest. Due to the paucity of biological material, we profiled POU6F1 genomic occupancy in mESCs rather than in embryos. Nevertheless, we identified many POU6F1-occupied promoters whose genes are involved in cell differentiation and embryogenesis, which strongly supports a model in which POU6F1 binds to chromatin and regulates gene transcription. Based on our results with mutant isoforms of *Dis3*, it appears that cytoplasmic DIS3 can rescue BL formation, which excludes a role for nuclear RNAs in triggering morula arrest and further supports a critical cytoplasmic function of DIS3 in degrading mRNAs. In addition, another RNA-exosome-associated RNase, EXOSC10, has also been indicated to regulate pre-implantation of mouse embryos, which further highlights the essential role of the RNA exosome complex in embryonic development.^32,33^

DIS3 ribonuclease mutations and altered protein levels have been reported in several human diseases.^20,21,24,34,35^ It is interesting that DIS3^D146N^ leads to 2-cell arrest of mouse embryos, whereas DIS3^ΔPIN^, a seemingly more dramatic deletion, does not affect pre-implantation development. We reason that DIS3^D146N^ has a strong dominant-negative effect by binding to substrates without degrading them, whereas DIS3^ΔPIN^ lacks the RNA-binding activity due to structural changes and has a less dominant-negative effect. The double mutations of DIS3^D146N+D487N^ could cause such a strong effect, and presumably, a gene-edited mouse model of DIS3^D146N+D487N^ would be embryonic lethal. In addition, the loss of cell division and increased cell size phenocopy, in part, transformed cells in human cancer, which may provide a possible mechanism of DIS3 mutations causing genomic instability, DNA repair defects, and genome rearrangement in human disease. Either DIS3 substrates or the RNA exosome-mediated transcription termination that are dysregulated by DIS3 knockout can directly affect local chromatin structure.^36^ Dysregulated genes (*MTAP*, *G3BP2*, *SEC23IP*, *USO1*) in homozygous *Dis3* null embryos mirror those found in the most common form of melanoma (GEO: GSE22301),^37^ which also has decreased levels of *Dis3*. Thus, our study not only elucidates the essential role of DIS3 ribonuclease in early embryonic development but also highlights its conserved roles across different cellular contexts in maintaining transcriptome integrity.

Collectively, our results demonstrate the essential role of DIS3 ribonuclease in mammalian early embryogenesis. We identified *Pou6f1* mRNA as a target of DIS3 that transcriptionally represses genes necessary to establish cell lineages. Insufficient degradation of *Pou6f1* leads to loss of cell differentiation and embryonic arrest. In summary, these results highlight a not heretofore reported regulatory role for DIS3 in regulating the abundance of POU6F1 to facilitate pre-implantation development in mammals.

### Limitations of the study

First, although persistent POU6F1 blocks cell fate specification in *Dis3* null embryos, there could be other factors dysregulated by *Dis3* knockout that contribute to the developmental arrest. Second, our study did not address a possible role of DIS3 homolog, DIS3L, in pre-implantation development. Third, a possible RNA-exosome-independent role of *Dis3* may also exist, which is beyond the scope of current study.

## STAR★METHODS

### RESOURCE AVAILABILITY

#### Lead contact

Further information and requests for resources, reagents and data will be addressed by the lead contact, Jurrien Dean (jurrien.dean@nih.gov ).

#### Materials availability

All reagents and resources generated in this study are available on request from the lead contact.

#### Data and code availability

Raw data, processed counts, and peaks of the single embryo RNA-seq the ChIP-seq for POU6F1 in mouse embryonic stem cells were deposited in the NCBI Gene Expression Omnibus (GSE201218). Processed differential analysis results are in supplemental tables. All scripts including R, Jupyter notebooks and Shell were uploaded to https://github.com/Di-aswater/public-scripts-Dis3Pou6f1EmbryoArrest. Any additional information, raw images and values are available from the lead contact upon request.

### EXPERIMENTAL MODEL AND SUBJECT DETAILS

#### Knockout mice generation

*Dis3* knockout mice were generated the same way as the *Dis3* floxed allele.^10^ Briefly, two guide RNAs (gRNAs) were designed to delete exons 3–5 (Table S1). The edited gene was repaired via non-homologous end-joining (NHEJ). The gRNAs (50 ng/μL) and *Cas9* mRNA (100 ng/μL) were mixed and microinjected into 1-cell mouse embryos. During microinjection, 1-cell embryos were collected from hormonally stimulated B6D2_F1_ female mice mated to B6D2_F1_ stud male mice into M2 medium (CytoSpring M2114). Embryos were treated with hyaluronidase (0.5 mg/mL, Sigma H4272) for 1 min at 37^◦^C to remove cumulus cells. Injected embryos were washed 10 times with KSOM (CytoSpring K0113) and cultured in KSOM at 37^◦^C with 5% CO_2_ overnight. 2-cell embryos were transferred into the oviducts of pseudopregnant ICR female mice at 0.5 days post coitus. Two *Dis3* knockout mouse lines were established with different DNA sequences between exon 2 and 6 after deleting exons 3–5 due to imprecise repair. Genotyping of the mice was performed using EmeraldAmp GT PCR Master Mix (Takara Bio RR310A). Oligos and genotyping primers are listed in Table S1.

#### Use of animals

All mice were maintained in compliance with the guidelines of the Animal Care and Use Committee of the National Institutes of Health under a Division of Intramural Research, NIDDK-approved animal study protocol. All experiments conformed to the regulatory standards.

#### *Ex vivo* culture of mouse embryos

For all *ex vivo* culture experiments, female mice at 8–12 wk old were stimulated with 5 IU of eCG for 48 h and 5 IU of hCG for 14 h and mated with male mice. Embryos were dissected in M2 medium, treated with hyaluronidase (0.5 mg/mL) for 1 min at 37^◦^C to remove cumulus cells. Embryos were washed 10 times with KSOM and cultured in KSOM at 37^◦^C with 5% CO_2_ until the desired developmental stage.

#### mESC derivation and directed differentiation

*Dis3^flox/flox^* female mice^10^ were stimulated sequentially with PMCG and hCG and mated to *Dis3^flox/flox^* male mice. Embryos were flushed from the uterus 3.5 days post coitus with pre-warmed ESGRO Complete Basal Medium (Sigma SF002). All wells of a 24-well plate were treated with 0.5 mL Gelatin solution (Sigma-Aldrich SF008) for 30 min at room temperature. After removing Gelatin, 0.5 mL pre-warmed ESGRO Complete Plus Clonal Grade Medium (Sigma SF001) was added into each well of the plate. Embryos at the blastocyst stage were transferred individually into each well. Embryos were cultured in ESGRO Complete Plus Clonal Grade Medium for 5 days at 37^◦^C with 5% CO_2_ and outgrowth of cells was observed from some embryos. Cells were treated with Accutase (Sigma-Aldrich SF006) at 37^◦^C for 1 min to dissociate into single cells. 1–5 single cells were transferred into fresh ESGRO Complete Plus Clonal Grade Medium, cultured, and maintained as a stable cell line. Cells were split when reaching 50% confluence and forming spheroids of 20–50 cells. For differentiation, cells were dissociated, washed with SF002 medium, and cultured in SF002 medium at 37^◦^C with 5% CO_2_ for 3 days. Transfection of mESCs is described in Method details below.

### METHOD DETAILS

#### Microinjection

The microinjection system consisted of a Zeiss inverted microscope, an Eppendorf Femtojet 4i Injector, a pair of Eppendorf Trans-ferman NK2 micro manipulators. The injector was set to auto mode, the injection time was 0.1 s, the compensation pressure was 15 and the injection pressure was 500. Needles were pulled from borosilicate glass with filament (BF150–75-10) using a Sutter Flaming/Brown Micropipette Puller. Injection solutions (2 μl) were loaded into the needle with an Eppendorf loading pipet Microloader (930001007) from the back. The holding pipets were Eppendorf VacuTip I (5195000036). Embryos were stored in pre-warmed M2 medium on a slide prior to and during microinjection.

#### Plasmid construction

The coding region of mouse *Dis3* gene was amplified from pcDNA3-mDIS3-FLAG, which was a gift from Dr. Zissimos Mourelatos (Addgene plasmid # 60046; http://n2t.net/addgene:60046; RRID: Addgene_60046).^40^ The T7 promoter sequence and the *Dis3* coding region were cloned into the vector pcDNA4-TO-Puromycin-mVenus-MAP, which was a gift from Dr. Dannel McCollum (Addgene plasmid # 44118; http://n2t.net/addgene:44118; RRID: Addgene_44118).^41^ The mutations of the *Dis3* gene were cloned using Gibson assembly.

The coding regions of selected DIS3 substrates, including *Pou6f1*, *Sox15*, *Efhd1*, *Pigl*, *Wdr45*, *Ovol2*, *Fbp1*, *Cacnb1*, *Cox6b2* and *Ninj2* were synthesized as dsDNA using gBlocks Gene Fragments (Integrated DNA Technologies, Inc). The T7 sequence and the synthesized fragments were cloned into pcDNA3.1-mCherry using Gibson assembly and their sequences are in Table S1.

#### Synthesis of mRNA

For mRNA synthesis from pcDNA4-TO-Puromycin-mVenus-MAP or pcDNA3.1 based plasmids, XbaI (NEB) was used to linearize the plasmid and DNA templates were purified with QIAquick PCR Purification Kit. RNA was synthesized with a mMESSAGE mMACHINE T7 ULTRA Transcription Kit (Thermo Fisher AM1345). In each 20 μL reaction, there were 2 μL of 10X T7 Reaction Buffer, 10 μL of 2X NTP/ARCA buffer, 2 μL of T7 enzyme, and 500 ng of linearized DNA template in nuclease free water. Reactions were mixed, incubated at 37^◦^C for 2 h and treated with 2 μL TURBO DNase. Reactions were polyadenylated per manufacturer’s guide. In each polyadenylation reaction, there were 20 μL 5X E-PAP buffer, 10 μL 25 mM MnCl_2_, 10 μL ATP solution, 36 μL nuclease free water, 4 μL E-PAP and 20 μL of the *in vitro* transcription reaction mix. Reactions were cultured at 37^◦^C for 45 min. Synthesized RNAs were purified with a MEGAclear Transcription Clean-Up Kit (Thermo Fisher AM1908).

#### Immunofluorescence staining and confocal microscopy of embryos

Embryos at desired stages were fixed (2% paraformaldehyde, 30 min, 37^◦^C). Embryos were washed twice with PBVT (PBS-3 mg/mL, polyvinylpyrrolidone-40, 0.1% Tween 20) and incubated with 0.5% Triton X-100 in PBS for 30 min. Embryos were blocked with 5% goat serum in PBVT for 1 h and incubated in primary antibodies overnight at 4^◦^C. On the second day, embryos were washed three times with PBVT and incubated with secondary antibodies overnight at 4^◦^C. On the third day, embryos were washed four times with PBVT, treated with Hoechst for 15 min and immediately transferred into 18-well dish (Ibidi 81817) for confocal microscopy. Images were obtained with a Zeiss LSM 780 microscope and processed with ImageJ Fiji software.

#### Single embryo RNA-seq

RNA-seq of single embryos was performed using GT-seq as described previously.^38^ Briefly, *Dis3*^*+/*−^ female mice were hormonally stimulated with PMSG followed by hCG and mated with *Dis3*^*+/*−^ male mice. Embryos were collected individually into 2.5 μL RLT (RNeasy) buffer at E2.5 and E2.75. 1 μL ERCC spike-in (1:100,000, ThermoFisher 4456740) was added to each sample. RNA was separated by oligo-dT conjugated beads, and the residual genomic DNA was used for genotyping of each embryo. In total, there were 6 wildtype, 2 heterozygous and 4 homozygous null embryos at the E2.5 stage, and 3 wildtype, 5 heterozygous and 3 homozygous null embryos at the E2.75 stage. RNA was reverse transcribed through template switch method and Superscript II reverse transcriptase (ThermoFisher 18064014). cDNA was amplified for 18 cycles and purified with Agencourt AMPure beads (A63881). Purified cDNA was evaluated with Agilent Bioanalyzer and made into sequencing libraries using the Nextera XT Sample Preparation Kit. RNA sequencing was performed at the NIDDK Genomic Core using HiSeq 2500.

#### RNA-seq analysis

RNA-seq raw reads were quality trimmed using Cutadapt (v3.4) with “-m 10 -j 8 -q 20,20 parameters”.^42^ Reads were mapped to GRCm38 using STAR (v2.7.8a) with default parameters to generate sorted BAM files.^43^ BAM files were counted by HTSeq (v0.11.4) with “-m intersection-strict -f bam -s no” parameters.^44^ Count files were used for differential analysis with DESeq2 to obtain differentially expressed genes.^45^ Differential analysis was performed individually at each stage and then two stages were merged by genotype to perform differential analysis between genotypes. Volcano plots were generated with Jupyter-notebook. All scripts were posted to Github (https://github.com/Di-aswater/public-scripts-Dis3Pou6f1EmbryoArrest).

#### Transfection of mESCs

mESC transfection was performed with Lipofectamine 2000 Transfection Reagent (ThermoFisher 11668027). Derived mESCs were treated with Accutase at 37^◦^C for 1 min to dissociate into single cells and washed with ESGRO Complete PLUS Clonal Grade Medium. Cells were transfected (500 μL per well) in a 24-well plate.

For each well, DNA (0.5 μg) was diluted with 50 μL ESGRO Complete PLUS Clonal Grade Medium. Lipofectamine 2000 (1 μL) was diluted with 50 μL ESGRO Complete PLUS Clonal Grade Medium and incubated for 5 min at room temperature. The diluted DNA and Lipofectamine 2000 were mixed and incubated for 5 min at room temperature. The transfection solution was added to cells and mixed thoroughly by rotating 10 times. Cells were cultured at 37^◦^C for 30 min, centrifuged at 1,300 rpm for 5 min, and dispensed into fresh ESGRO Complete PLUS Clonal Grade Medium.

#### Immunofluorescence staining and confocal microscopy of mESCs

Cells were fixed using 4% paraformaldehyde (30 min, room temperature). Cells were washed twice with PBS and permeabilized with 0.5% Triton X-100 for 30 min. Cells were blocked with 5% goat serum diluted in PBS-0.1% Tween 20 for 1 h at room temperature. Cells were incubated with primary antibodies overnight at 4^◦^C. On the second day, cells were washed with PBS-0.1% Tween 20 for 20 min X4 and incubated with secondary antibodies. On the third day, cells were washed with PBS-0.1% Tween 20 for 20 min X4, incubated with DAPI and imaged using a Zeiss LSM 780 confocal microscope. At each step, cells were collected by centrifuging at 1300 rpm for 5 min.

#### Quantitative RT-PCR of mESCs

Cells were washed with PBS and collected in RLT (RNeasy) buffer. Total RNA was extracted with a Qiagen RNeasy Plus Micro Kit (74,034). Total RNA was used for reverse transcription with a ProtoScript First Strand cDNA Synthesis Kit (New England Biolabs E6300S) in a 20 μL reaction. For each reaction, there was 1 μg total RNA and 2 μL d(T)_23_VN oligos (50 μM) in nuclease free water (8 μL total). The mixture was denatured for 5 min at 70^◦^C, spun down briefly and put on ice. In each reverse transcription reaction, there was 10 μL AMV Reaction Mix, 2 μL AMV Enzyme Mix, and 8 μL RNA mix prepared as described above. Reactions were incubated at 25^◦^C for 5 min, 42^◦^C for 1.5 h and 80^◦^C for 5 min. The generated cDNA was diluted to 50 μL by adding 30 μL nuclease free water. qRT-PCR was performed with iTaq Universal SYBR Green Supermix (Bio-Rad Laboratories 1725122). 10 μL reactions were performed in a MicroAmp Optical 384-Well Reaction Plate with Barcode (4309849). In each reaction, there were 5 μL 2X Supermix, 0.25 μL each primer (10 μM), 0.5 μL DNA (1–10 ng/μL) and 4 μL water. qPCR reactions were performed using QuantStudio 6 Flex Real-Time PCR System (Thermo Fisher Scientific).

#### ChIP-seq

mESCs were transfected with pCAG-mCherry or pCAG-pou6f1-mCherry. Each transfection sample was performed using 15 μg DNA and 30 μL Lipofectamine 2000 (details described above) in a T75 cell culture flask of about 1,000,000 cells. Each sample had three biological replicates. Cells were dissociated by Accutase and collected into lobind tubes 48 h after transfection. ChIP-seq libraries were prepared using True MicroChIP & MicroPlex Library Preparation Package (C01010131) according to the manufacturer’s instructions. Briefly, cells were washed with PBS and fixed by 1% formaldehyde (15 min, room temperature). Crosslinking was terminated by adding glycine (115 μL per 1 mL reaction) and incubated for 5 min at room temperature. Cells were washed twice with HBSS (ThermoFisher 14170112), collected, and frozen overnight at −80^◦^C. On the second day, cells were lysed and sonicated using the Bioruptor for 10 cycles. Sheared DNA was purified using phenol/chloroform/isoamyl alcohol (25:24:1) (Sigma-Aldrich P2069). 20 μL from each sample was removed as the input sample and held at −80^◦^C. 1 μg anti-mCherry antibody (Abcam Ab167453) or rabbit IgG was added into each sample. Samples were incubated overnight at 4^◦^C. Pre-treated magnetic beads were added to each sample and incubated for 2 h at 4^◦^C. Samples were washed, de-crosslinked and purified using phenol/chloroform/isoamyl alcohol (25:24:1). Quality of the samples was evaluated by Bioanalyzer 2100. Samples were used for library preparation following the MicroPlex Library Preparation manual, purified with Agencourt AMPure XP beads, and pooled for sequencing. The sequencing was performed at the NIDDK Genomics Core using HiSeq 2500.

#### ChIP-seq analysis

Original ChIP-seq fastq files were aligned to GRCm38 using Bowtie2 (v2.4.4).^46^ Generated SAM files were converted to BAM files, sorted, and filtered for uniquely mapping reads using Sambamba (v0.8.1).^47^ Peaks were called using MACS2 (v2.2.7.1),^48^ and the anti-IgG sample and anti-mCherry of mCherry transfected samples used as controls, respectively. Each biological replicate was processed individually to obtain peaks. Consensus peaks were generated using BEDtools (v2.30.0).^49^ When plotting heatmaps, coverage values (CPM normalized) were calculated from BAM files using deepTools (v3.5.1),^50^ and processed with computeMatrix and plotHeatmap functions of deepTools.

#### Electrophoretic mobility shift assay (EMSA)

*Pou6f1* probe was synthesized by *in vitro* transcription. The *Pou6f1* exon 8 was selected as the probe because of the DIS3 occupancy in the PAR-CLIP seq result.^9^ The probe was 121 nt long (Table S1), and its template was synthesized as ssDNA by adding T7 in the 5^′^ end and T3 in the 3^′^ end (Table S1). The ssDNA was PCR amplified by T7 and T3 primers. Purified dsDNA was used as the template for *in vitro* transcription using MEGAscript T7 Transcription Kit (ThermoFisher AM1333). For unlabeled probe synthesis, each reaction had 2 μL 75 mM ATP, GTP, CTP, UTP, respectively, plus other essential components per manufacture’s user guide. For Biotin-labeled probe synthesis, each reaction had 2 μL 75 mM ATP, 2 μL 75 mM GTP, 1.5 μL 75 mM CTP, 1.5 μL 75 mM UTP, 3.75 μL 10 mM Bio-11-CTP (ENZO ENZ-42818), 3.75 μL 10 mM Bio-11-UTP (Thermo Scientific AM8450) plus other essential components. Synthesized probes were purified by Ambion RNAqueous columns (Invitrogen AM1912).

To prepare the crude cell extract, pcDNA4-TO-Dis3ΔNLS-D146N-D487N-mVenus plasmid was transfected into the derived mESCs and cultured for 48 h. Cells were collected, washed and transferred to newly prepared lysis buffer (150 mM NaCl, 1% Triton X-100, 50 mM Tris-Cl, Protease inhibitor cocktail, pH 7.4). Cell-lysis solution was mixed by vortex and incubated on ice for 30 min. Lysis was frozen at −80^◦^C overnight, thawed, diluted to 1 mg/mL, and centrifuged at 12,000 rpm for 10 min to remove debris before use. EMSA was performed using LightShift Chemiluminescent RNA EMSA Kit (Thermo Scientific 20,158). DNA Retardation Gels (6%) was used (Thermo Scientific EC6365BOX). In each 20 μL reaction, there were 1 μL RNase inhibitor (ThermoFisher), 2 μL 10X binding buffer, 1 μL 50% glycerol, 1 μL 100 mM MgCl_2_,1 μL 1 ng/μL bio-probe, 2 mL 0.1 ng/μL unlabeled probe (when necessary), 1 μL protein extract and 1 μL anti-FLAG (Sigma F1804) in ultrapure water. Probes were denatured by incubation at 80^◦^C for 5 min, spun down and put on ice right before use. Reactions were incubated at room temperature for 30 min. 5 μL 5X loading buffer was added to each 20 μL reaction and mixed by pipetting. 20 μL of reaction was loaded onto each well for electrophoresis through 6% non-denaturing polyacrylamide gel in 0.5X TBE buffer, transferred to a nylon membrane (Thermo Scientific AM10100), crosslinked to the membrane by a commercial UV-light crosslinking instrument (120 mJ/cm^2^ for 60 s). Membrane was incubated by the blocking buffer, washing buffer, substrate equilibration buffer and detected per user’s guide.

### QUANTIFICATION AND STATISTICAL ANALYSIS

Statistical analyses in Figures 1–3 were performed using two-tailed Student’s t-test. P values are labeled in each figure and the figure legends. All group photos were from one assay out of at least three repeated assays. All single embryo photos were the most representative picture from all repeated experiments. The number of samples/embryos is labeled in each figure or figure legend. In the RNA-seq analysis, P-adj <0.1 and log_2_ fold change >1 was implemented to filter the significantly changed genes.

## Supplementary Material

1

2

3

4

5

## Figures and Tables

**Table:** KEY RESOURCES TABLE

REAGENT or RESOURCE	SOURCE	IDENTIFIER
Antibodies		

Anti-mCherry	Abcam	Ab167453; RRID:AB_2571870
Anti-E-cadherin	Cell Signaling	3195S; RRID:AB_2291471
Anti-NANOG	Abcam	Ab80892; RRID:AB_2150114
Anti-CDX2	Abcam	Ab76541; RRID:AB_1523334
Anti-OCT4	Abcam	Ab19857; RRID:AB_445175
Anti-DIS3	Abcam	Ab223767
Anti-POU6F1	Lifespan Biosciences	LS-C482796-100
Anti-SSEA-1	Abcam	Ab16285; RRID:AB_870663
Anti-NESTIN	R&D Systems	MAB2736-SP
Goat anti-rabbit IgG (H + L) cross-adsorbed secondary antibody, Alexa Fluor 546	Invitrogen	A-11010
Goat anti-mouse IgG (H + L) cross-adsorbed secondary antibody, Alexa Fluor 647	Invitrogen	A-21235
Goat anti-rabbit IgG (H + L) cross-adsorbed Secondary Antibody, Alexa Fluor 488	Invitrogen	A-11008
Goat anti-Mouse IgG (H + L) cross-adsorbed Secondary Antibody, Alexa Fluor 488	Invitrogen	A-11001
Anti-cleaved caspase3	Cell Signaling Technology	9661s; RRID:AB_2341188
Anti-FLAG	Sigma	F1804; RRID:AB_262044

Chemicals, peptides, and recombinant proteins		

Paraformaldehyde	Electron Microscopy Sciences	50-980-492
Goat serum	Sigma-Aldrich	G9023-10ML
Phenol/chloroform/isoamyl alcohol (25:24:1)	Sigma-Aldrich	P2069
Phalloidin	ThermoFisher	A12379
Tween 20	Sigma-Aldrich	P1379-25ML
DAPI	Sigma-Aldrich	D9542-1MG
Triton X-100, 10%	ThermoFisher	85111
BSA	MP Biomedicals	0216006910
Hyaluronidase from bovine testes	Sigma-Aldrich	H4272-30mg
DTT	Sigma-Aldrich	10197777001
Mineral oil	Sigma-Aldrich	M5310-1L
PVP-40	Sigma-Aldrich	PVP40-100G
Bio-11-CTP	ENZO	ENZ-42818
Bio-11-UTP	Thermo Scientific	AM8450
SUPERase$ In^™^ RNase Inhibitor (20 U/mL)	ThermoFisher	AM2694
Novex^™^ TBE Running Buffer	Thermo Scientific	LC6675
Gelatin	Sigma-Aldrich	SF008

Critical commercial assays		

Alkaline Phosphatase Staining Kit II	Biotrend	00-0055
Lipofectamine 2000 Transfection Reagent	ThermoFisher	11668027
RNeasy Plus Micro Kit	Qiagen	74034
ProtoScript First Strand cDNA Synthesis Kit	New England Biolabs	E6300S
iTaq^™^ Universal SYBR^®^ Green Supermix	Bio-Rad Laboratories	1725122
MicroAmp^™^ Optical 384-Well Reaction Plate	ThermoFisher	4309849 with Barcode
MicroAmp^™^ Optical Adhesive Film	ThermoFisher	4360954
True MicroChIP & MicroPlex Library Preparation^™^ Package	Diagenode	C01010131
mMESSAGE mMACHINE^®^ T7 ULTRA Transcription Kit	ThermoFisher	AM1345
MEGAshortscript^™^ T7 Transcription Kit	ThermoFisher	AM1354
MEGAclear^™^ Kit	ThermoFisher	AM1908
RNeasy Protect Mini Kit	Qiagen	74124
RNase-Free DNase Set (50)	Qiagen	79254
SuperScript^™^ II Reverse Transcriptase	ThermoFisher	18064014
QuickExtract^™^ DNA Extraction Solution	Epicentre	QE09050
HBSS	ThermoFisher	14170112
EmeraldAmp^®^ GT PCR Master Mix	Takara Bio	RR310A
MEGAscript^™^ T7 Transcription Kit	ThermoFisher	AM1333
RNAqueous^™^ Total RNA Isolation Kit	Invitrogen	AM1912
LightShift^®^ Chemiluminescent RNA EMSA Kit	Thermo Scientific	20158
ERCC RNA Spike-In Mix	ThermoFisher	4456740

Deposited data		

Raw and processed RNA-seq data	This study	GSE201218
Raw and processed ChIP-seq data	This study	GSE201218

Experimental models: Cell lines		

*Dis3* knockout mice	This study	N/A
*Dis3* flox/flox mice and derived mouse embryonic stem cell lines	This study	N/A

Oligonucleotides		

All primers used for genotyping and qRT-PCR (Table S1)	This study	N/A
All sgRNAs for CRISPR/Cas9 (Table S1)	This study	N/A
Template switch oligo	Macaulay et al., 2016^38^	N/A
*Dis3* morpholino (Table S1)	This study (Gene Tools)	N/A
*Dis3 specific* control morpholino (Table S1)	This study (Gene Tools)	N/A
*Pou6f1* morpholino (Table S1)	This study (Gene Tools)	N/A
Control morpholino	Gene Tools	Random Control Oligo 25-N
*Pou6f1* siRNA	Thermo Scientific	s71995
*Control* siRNA	Thermo Scientific	Silencer ^®^Select Negative Control #1 siRNA

Recombinant DNA		

pCAG-mCherry	This study	N/A
pCAG-Pou6f1-mCherry	This study	N/A
pcDNA3.1-mVenus	This study	N/A
pcDNA3.1-mCherry	This study	N/A
pcDNA4-TO-Dis3cds-mVenus-puro	This study	N/A
pcDNA4-TO-Dis3D146N-mVenus	This study	N/A
pcDNA4-TO-Dis3D487N-mVenus	This study	N/A
pcDNA4-TO-Dis3D146N-D487N-mVenus	This study	N/A
pcDNA4-TO-Dis3ΔPIN-mVenus	This study	N/A
pcDNA4-TO-Dis3ΔNLS-mVenus	This study	N/A
pcDNA4-TO-Dis3ΔNLS-D146ND487N-mVenus	This study	N/A
pcDNA3.1-Pou6f1-mCherry	This study	N/A
pcDNA3.1-Sox15-mCherry	This study	N/A
pcDNA3.1-Cox6b2-mCherry	This study	N/A
pcDNA3.1-Efhd1-mCherry	This study	N/A
pcDNA3.1-Ovol2-mCherry	This study	N/A
pcDNA3.1-Ninj2-mCherry	This study	N/A
pcDNA3.1-Pigl-mCherry	This study	N/A
pcDNA3.1-Cacnb1-mCherry	This study	N/A
pcDNA3.1-Fbp1-mCherry	This study	N/A
pcDNA3.1-Wdr45-mCherry	This study	N/A
pcDNA3.1-Pou6f1-mCherry-3UTR	This study	N/A
pCAG-Cre-IRES2-GFP	Addgene Woodhead et al., 2006^39^	# 26646
pcDNA3-mDIS3-FLAG	Addgene Liu et al., 2014^40^	# 60046
pcDNA4-TO-Puromycin-mVenus-MAP	Addgene Ma et al., 2012^41^	# 44118

Software and algorithms		

ImageJ Fiji	ImageJ Fiji	N/A
Python 3	Python Foundation	N/A
RStudio	RStudio	N/A
Cutadapt (v3.4)	NBIS (National Bioinformatics Infrastructure Sweden) Martin, 2011^42^	N/A
STAR (v2.7.8a)	Dobin et al., 2013^43^	N/A
HTSeq (v0.11.4)	Anders et al., 2015^44^	N/A
DESeq2	Love et al., 2014^45^	N/A
Bowtie2 (v2.4.4)	Langmead, et al., 2012^46^	N/A
Sambamba (v0.8.1)	Tarasov et al., 2015^47^	N/A
MACS2 (v2.2.7.1)	Zhang et al., 2008^48^	N/A
BEDtools (v2.30.0)	Quinlan and Hall, 2010^49^	N/A
deepTools (v3.5.1)	Ramírez et al., 2016^50^	N/A
IDR (v2.0.3)	Li, et al., 2011^51^	N/A
ChIPseeker (v1.20.0)	Yu et al., 2015^52^	N/A
EnsDb.Mmusculus.v79 (v2.99.0)	Rainer et al., 2019^53^	N/A
clusterProfiler (v3.12.0)	Wu et al., 2021, Yu et al., 2012^54,55^	N/A
AnnotationDbi	(Pagès H, Carlson M, Falcon S, Li N, 2021)	N/A
TxDb.Mmusculus.UCSC.mm10.knownGene	(Team BC, Maintainer BP 2019)	N/A
org.Mm.eg.db	(Carlson M, 2019)	N/A

Other		

ESGRO Complete Plus Clonal Grade Medium	Sigma-Aldrich	SF001-500P
ESGRO Complete Basal Medium	Sigma-Aldrich	SF002-500
ESGRO Complete Freezing Medium	Sigma-Aldrich	SF005
Accutase^™^ Cell Dissociation Solution	Sigma-Aldrich	SF006
ESGRO Complete Gelatin (ready to use)	Sigma-Aldrich	SF008
EmbryoMax^®^ D-PBS, w/o Ca^2+^ & Mg^2+^	Sigma-Aldrich	BSS-1006-B
EmbryoMax^®^ 0.1% Gelatin Solution	Sigma-Aldrich	ES-006-B
Agencourt AMPure beads	ThermoFisher	A63881
M2 medium	CytoSpring	M2114
KSOMAA with BSA and Phenol red	CytoSpring	K0113
Zenodo archive of GitHub repository	https://doi.org/10.5281/zenodo.7506639	N/A

## Highlights

- Homozygous *Dis3* knockout mouse embryos arrest as morulae in early development
- DIS3 ribonuclease degrades *Pou6f1* mRNA to de-repress embryonic cell differentiation
- POU6F1 globally occupies promoters of genes to regulate transcription
- Mutations of DIS3 result in cell exclusion in pre-implantation development
