# Supplementary material for: RNA exosome ribonuclease DIS3 degrades Pou6f1 to promote mouse pre-implantation cell differentiation
Source: Cell Rep. Author manuscript; Available in PMC 2023 Oct 23. (PMC10387129; doi:10.1016/j.celrep.2023.112047)
Supplement: 1 [file NIHMS1878915-supplement-1.pdf]

Cell Reports, Volume *42*

## Supplemental information

# RNA exosome ribonuclease DIS3 degrades *Pou6f1* to promote mouse pre-implantation cell differentiation

**Di Wu and Jurrien Dean**

## SUPPLEMENTAL INFORMATION

**Figure S1.** *Dis3* null embryos arrest at the morula stage.

**Figure S2.** Immunostaining of *Dis3* null embryos for apoptosis, endomembrane integrity and differentiation.

**Figure S3.** Identification of *Pou6f1* from single embryo RNA-seq.

**Figure S4.** Overexpression of selected DIS3 substrates does not phenocopy *Dis3* null embryos.

**Figure S5.** *Pou6f1* knockdown can partially rescue the *Dis3* null embryos.

**Figure S6.** Identification of POU6F1 occupied genes using ChIP-seq of mESCs.

## SUPPLEMENTAL FIGURES

### Figure S1

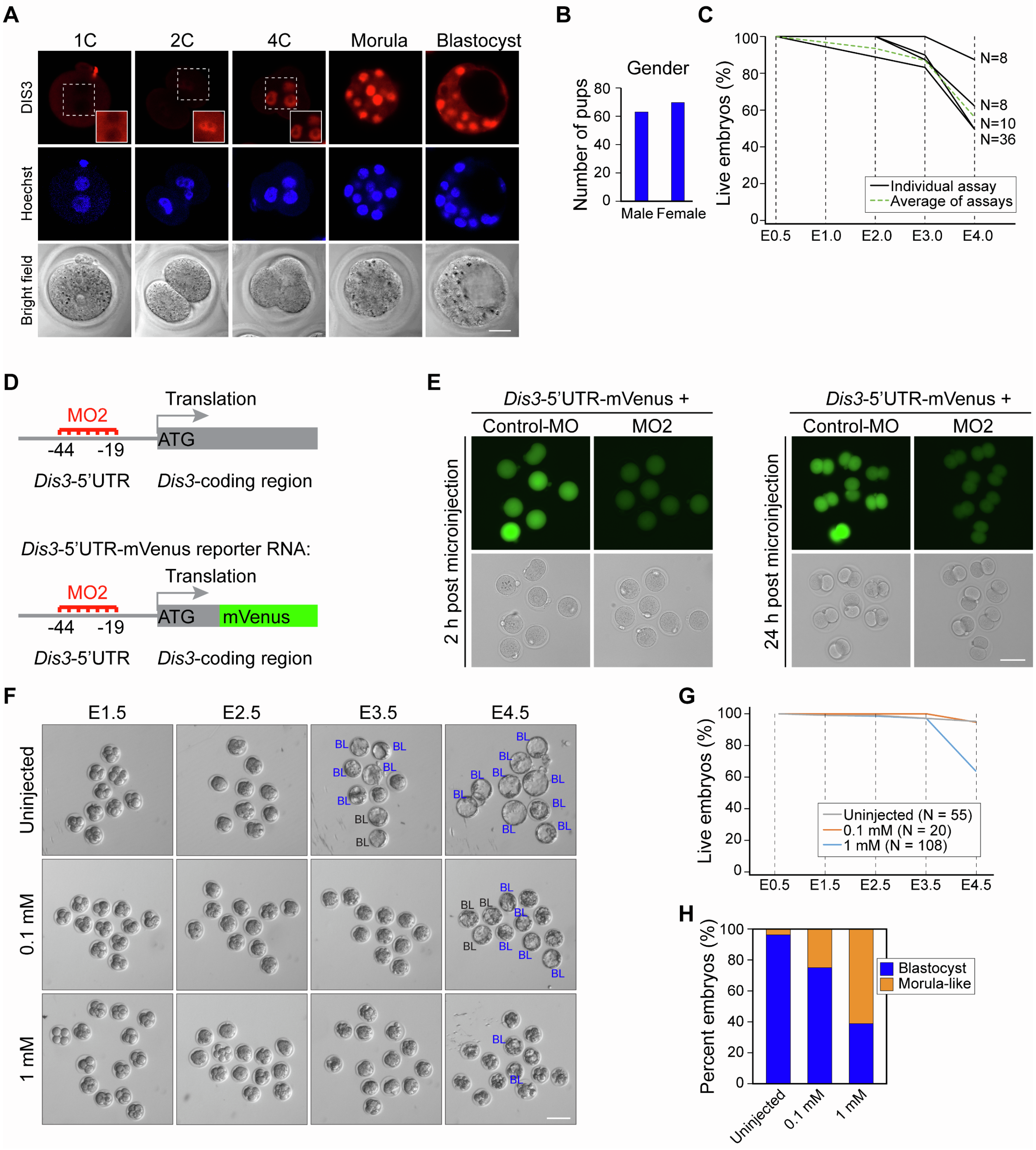


**Figure S1. *Dis3* null embryos arrest at the morula stage.**

(A) Immunofluorescence of DIS3 in wildtype embryos at 1C, 2C, 4C, morula and blastocyst stages. In 1C, 2C and 4C stages, the dashed line-region is contrast-enhanced in the DIS3 channel and shown in the inset.

(B) Bar graph showing gender distribution of the pups born from *Dis3* heterozygous mating.

(C) Line graph showing the viability of embryos derived from *Dis3* heterozygous mating during pre-implantation development. Black lines: four assays; green dashed line: average of the four assays.

(D) Schematic of *Dis3* MO2 (morpholino 2) in blocking *Dis3* mRNA translation (top) and schematic of the reporter RNA to test MO2 efficiency (bottom).

(E) Fluorescent and bright field images of wildtype embryos co-injected with reporter RNA (*Dis3-mVenus*) and control MO (scrambled) or *Dis3*-specific MO2 at 2 h (left) and 24 h (right) post-injection.

(F) Bright field live imaging of wildtype embryos microinjected with *Dis3* MO2. BL, blastocyst.

(G and H) Line graph and bar graph showing the survival rate of *Dis3* MO2-injected embryos and the stage of embryos at E3.5.

Scale bars, 20 μm in A, 100 μm in E, F.

# Figure S2

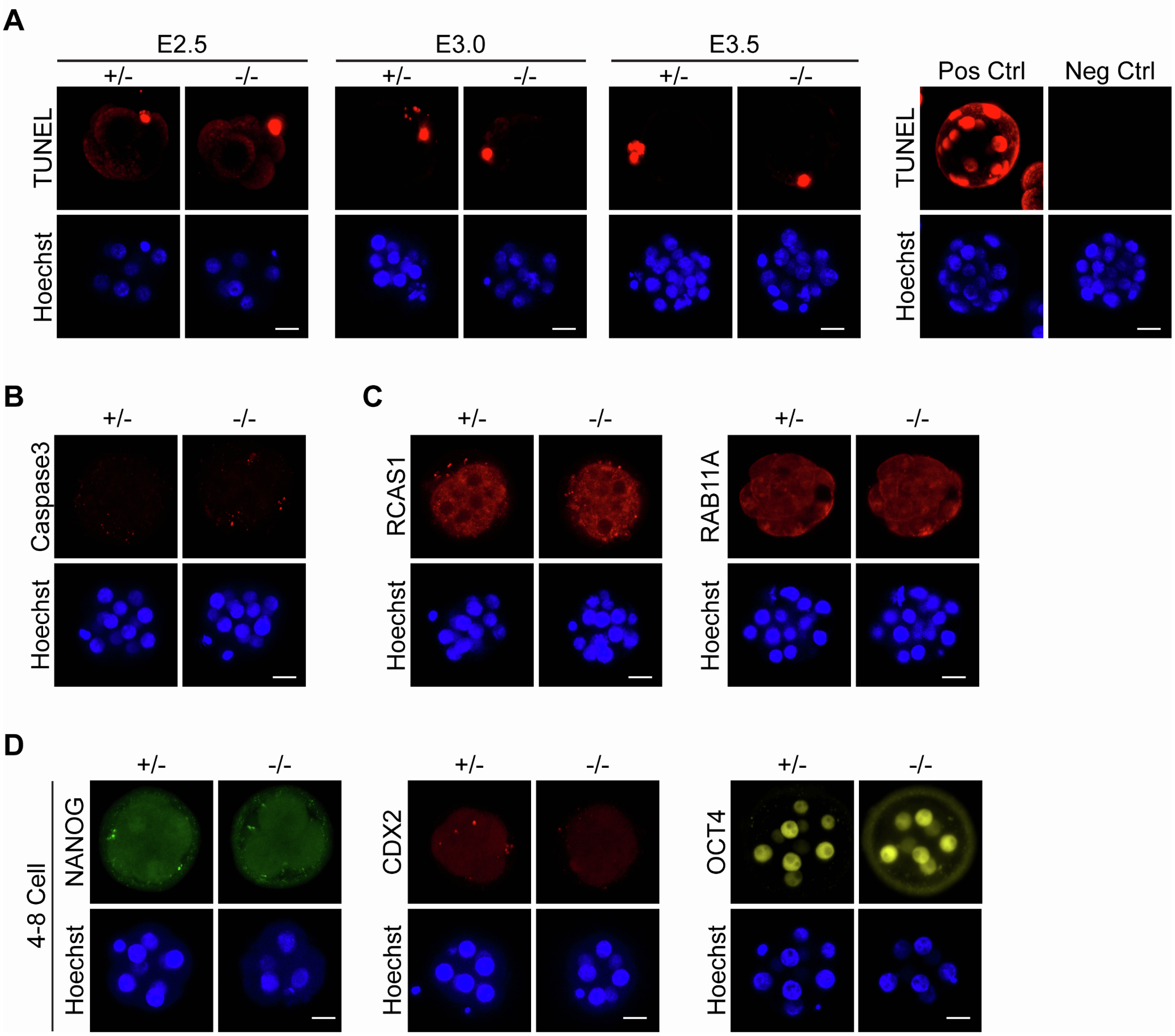


**Figure S2. Immunostaining of *Dis3* null embryos for apoptosis, endomembrane integrity and differentiation.**

(A) Immunofluorescent TUNEL assay of *Dis3* heterozygous (+/-) and homozygous (-/-) null embryos. Pos Ctrl: positive control of embryos treated with DNase I (3 U/$\mu$L). Neg Ctrl: negative control of embryos without staining.

(B and C) Immunofluorescence of heterozygous and homozygous null *Dis3* embryo after staining with antibodies to cleaved caspase 3, RCAS1 and RAB11A.

(D) Same as (B) but after staining with antibodies to NANOG, CDX2 and OCT4 at the 4-8 cell stage.

Scale bars, 20 $\mu$m.

## Figure S3

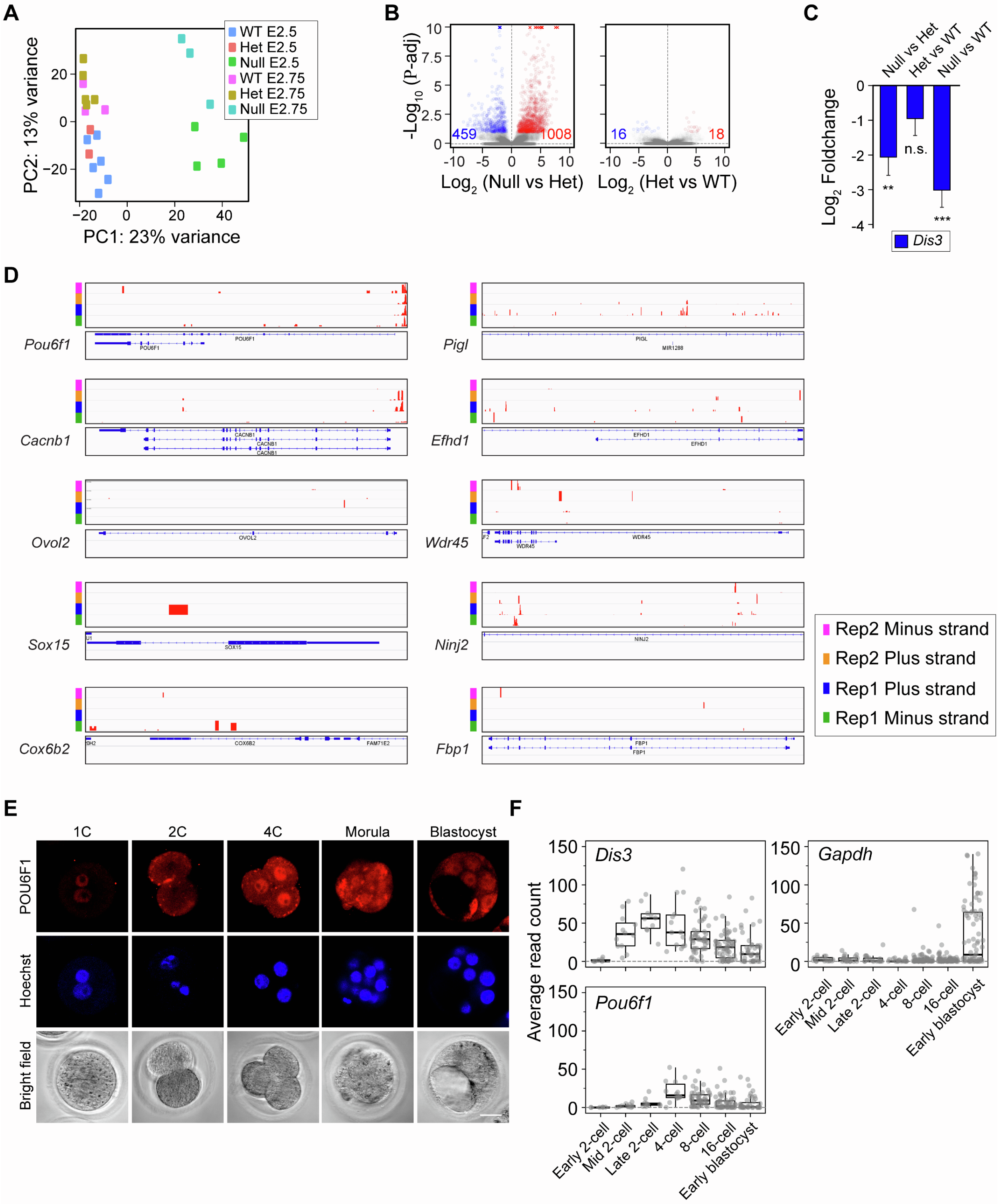
A
WT E2.5
Het E2.5
Null E2.5
WT E2.75
Het E2.75
Null E2.75
PC2: 13% variance
PC1: 23% variance
B
-Log10 (P-adj)
459
1008
16
18
Log2 (Null vs Het)
Log2 (Het vs WT)
C
Null vs Het
Het vs WT
Null vs WT
Log2 Foldchange
**
n.s.
***
Dis3
D
Pou6f1
Pigl
Cacnb1
Efhd1
Ovol2
Wdr45
Sox15
Ninj2
Cox6b2
Fbp1
Rep2 Minus strand
Rep2 Plus strand
Rep1 Plus strand
Rep1 Minus strand
E
1C
2C
4C
Morula
Blastocyst
POU6F1
Hoechst
Bright field
F
Dis3
Gapdh
Pou6f1
Average read count
Early 2-cell
Mid 2-cell
Late 2-cell
4-cell
8-cell
16-cell
Early blastocyst

**Figure S3. Identification of *Pou6f1* from single embryo RNA-seq.**

(A) Principal component analysis (PCA) of RNA-seq samples from single heterozygous or homozygous null embryo at E2.5 or E2.75.

(B) Volcano plots of differentially expressed promoter upstream transcript (PROMPTs) in single embryo RNA-seq of combined E2.5 and E2.75 stages.

(C) Bar graph of changes in abundance of *Dis3* transcript in RNA-seq of wildtype (WT), heterozygous (Het) or homozygous (Null) null $Dis3^{cKO}$ embryos. **, P-adj<0.01, ***, P-adj<0.001, n.s., not significant.

(D) Integrated genomic viewer of PAR CLIP-seq results of 10 substrate genes. Reference used is the human genome. The four tracks shown include two replicates of plus strand and minus strands.

(E) Immunofluorescence staining of POU6F1 and Hoechst in wildtype embryos at 1C, 2C, 4C, morula and blastocyst stages.

(F) Average read counts of *Dis3, Pou6f1* and *Gapdh* from GSE45719 single cell RNA-seq from 2-cell stage to blastocyst stage.

Scale bar, 20 μm.

# Figure S4

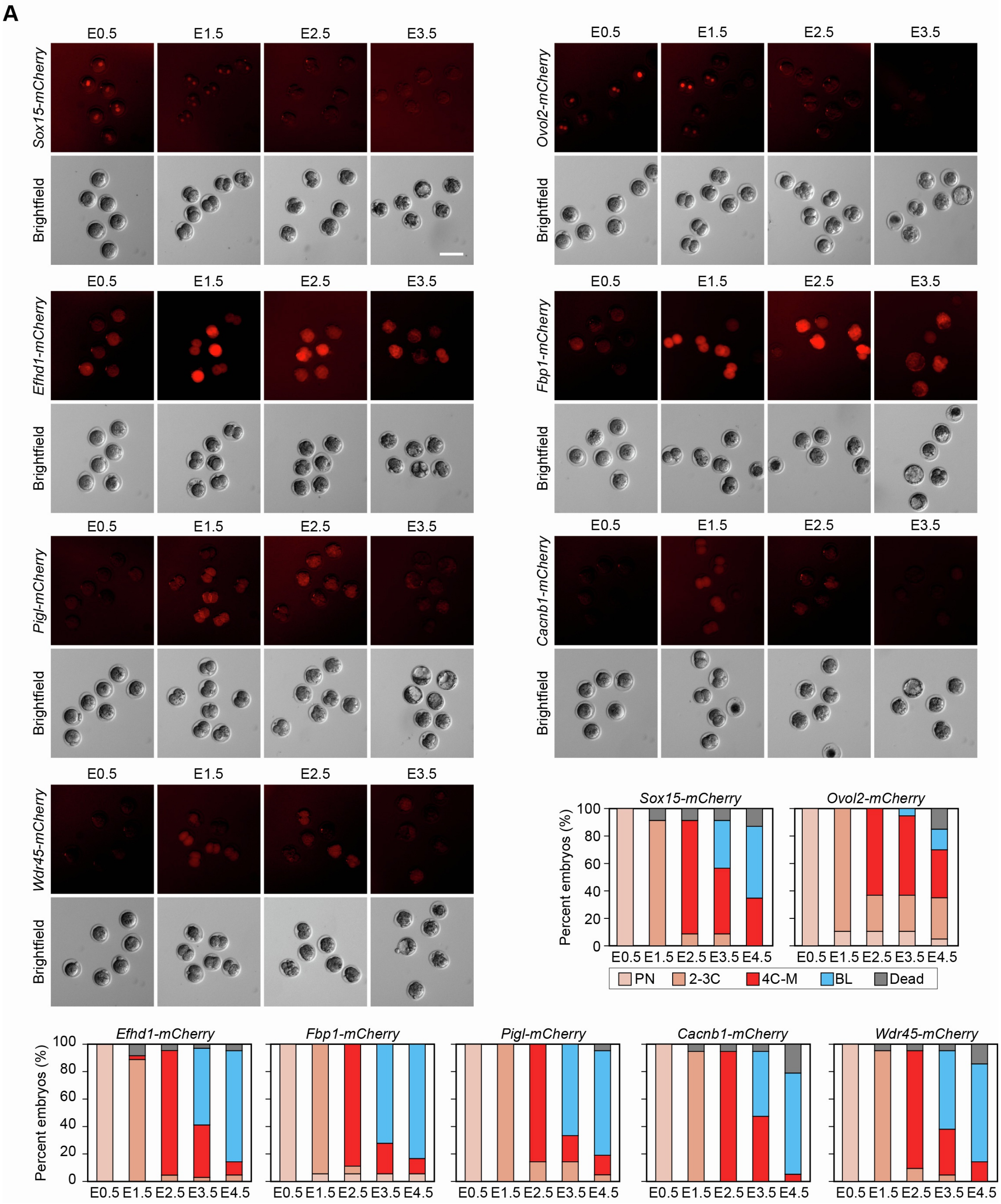
A
E0.5
E1.5
E2.5
E3.5
Sox15-mCherry
Ovol2-mCherry
Efhd1-mCherry
Fbp1-mCherry
Pigl-mCherry
Cacnb1-mCherry
Wdr45-mCherry
Brightfield
Percent embryos (%)
0
20
40
60
80
100
E4.5
PN
2-3C
4C-M
BL
Dead

**Figure S4. Overexpression of selected DIS3 substrates does not phenocopy *Dis3* null embryos.**

Fluorescence and bright field imaging of wildtype embryos injected with potential DIS3 substrates individually: *Sox15-mCherry* (N=23), *Ovol2-mCherry* (N=26), *Efhd1-mCherry* (N=22), *Fbp1-mCherry* (N=22), *Pigl-mCherry* (N=21), *Cacnb1-mCherry* (N=19), *Wdr45-mCherry* (N=21). Bar graph showing the ratio of embryos at each stage from E0.5 to E4.5 after *ex vivo* culture. Scale bar, 100 $\mu$m.

## Figure S5

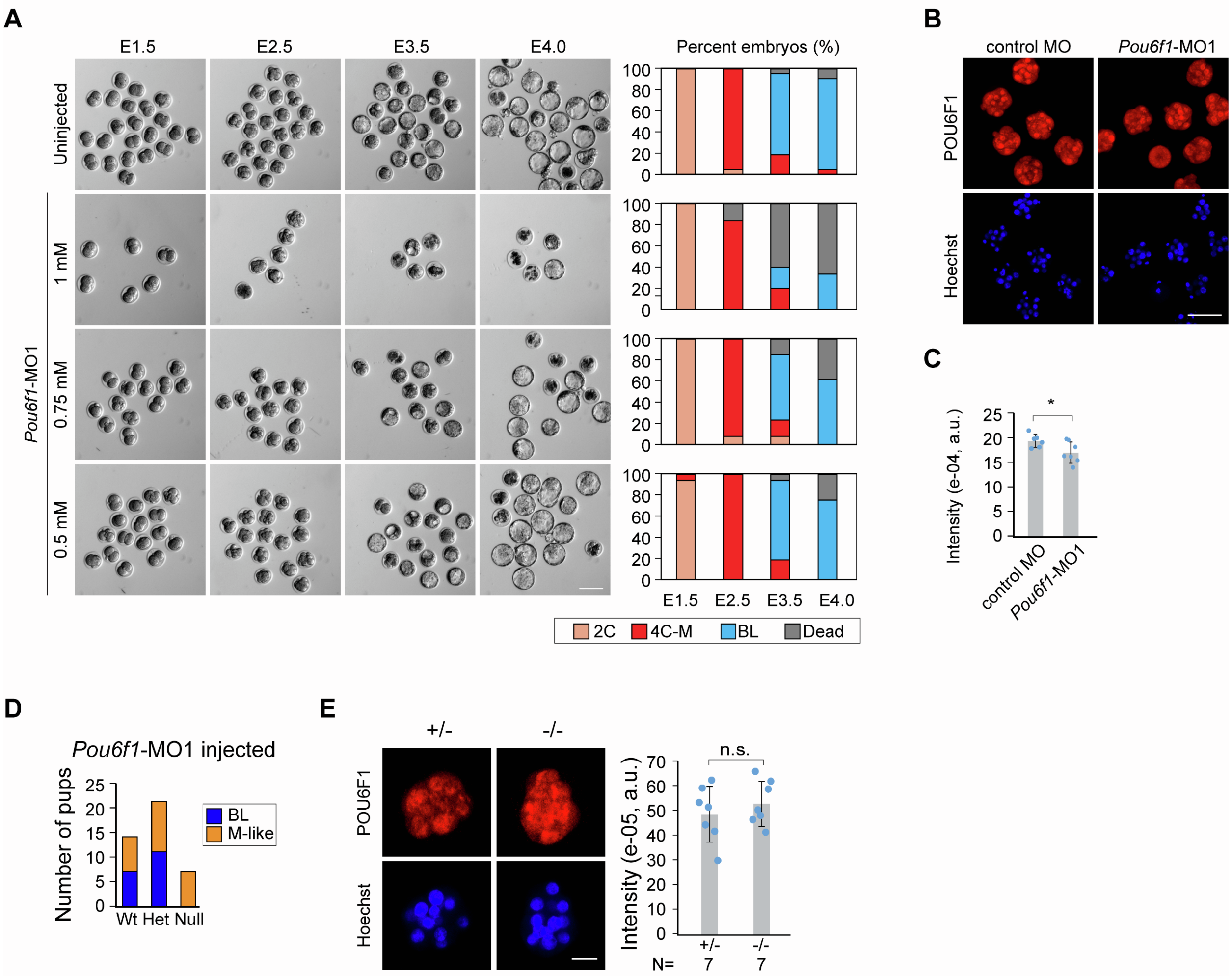


**Figure S5. *Pou6f1* knockdown can partially rescue the *Dis3* null embryos.**

(A) Microinjection of 0.5, 0.75 and 1 mM of *Pou6f1* MO1 into wildtype embryos. Embryos are injected or uninjected and cultured to E4.0. The developmental progression is recorded on the right.

(B and C) POU6F1 immunofluorescence and quantification of wildtype embryos that are microinjected with control MO or *Pou6f1* MO1. Embryos are at E3.0.

(D) Number of BL and M-like embryos in *ex vivo* culture at E3.5 of embryos derived from *Dis3* heterozygous mating and microinjected with *Pou6f1* MO1 at 1-cell stage.

(E) POU6F1 immunofluorescence and quantification of *Dis3* heterozygous (+/-) and homozygous (-/-) null embryos. n.s., not significant. Bar graph shows embryos obtained from one assay out of three biological replicates.

## Figure S6

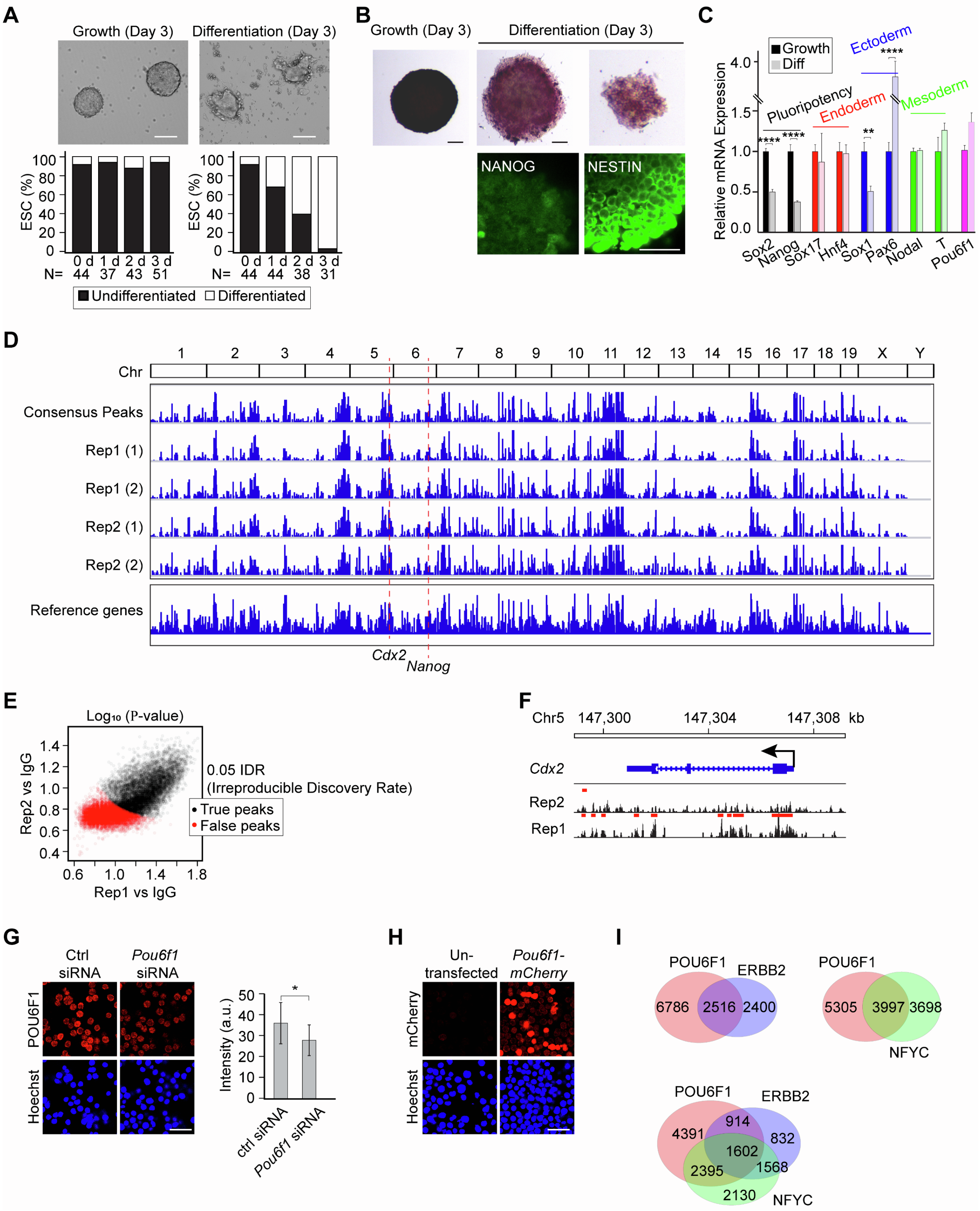


**Figure S6. Identification of POU6F1 occupied genes using ChIP-seq of mESCs.**

(A) Brightfield images of derived mESCs cultured in media with self-renewal factors (left) or basal media without self-renewal factors (right). Bar graphs (below) show the percent of colonies with undifferentiated or differentiated morphology. Scale bars, 100 μm.

(B) Differentiation of mESC labeled with alkaline phosphate (AP) and immunofluorescence after staining with antibodies to NANOG and NESTIN (lower panels). Scale bar, 50 μm.

(C) Bar plot of quantitative RT-PCR of different marker transcripts in growth and differentiation (Diff) conditions of derived mESCs. ****, Sox2, Nanog, Pax6, $P<0.001$; **, Sox1, $P<0.01$; two-tailed Student's t-test.

(D) Integrated genomic viewer of ChIP-seq results of two biological replicates. For each replicate (Rep1 and Rep2), peaks are called either against IgG of the *Pou6f1-mCherry* transfected cells, Rep1 (1) and Rep2 (1), or against mCherry of the *mCherry* transfected cells Rep1 (2) and Rep2 (2).

(E) Dot plot showing IDR (irreproducible discovery rate) analysis. Each dot is a peak from two replicates (Rep1 and Rep2) called against IgG. Black dots are IDR value less than 0.05, which indicates true reproducible peaks; red dots are IDR values larger than 0.05, which are indicate false peaks.

(F) POU6F1 peaks on *Cdx2*. Red bars: peaks called against IgG samples in Rep1 and Rep2.

(G) POU6F1 immunofluorescence and quantification in derived mouse embryonic stem cells (mESC) after control siRNA (Ctrl siRNA) or *Pou6f1* siRNA transfections. *, 0.03.

(H) mCherry fluorescence of untransfected mESC and mESC after *Pou6f1-mCherry* transfection.

(I) Venn diagrams showing binding of POU6F1 and ERBB2 (left), POU6F1 and NFYC (middle), POU6F1, ERBB2 and NFYC (right) to common (2516, 3997, 1602, respectively) genes.
